# Bacterial F-type ATP synthases follow a well-choreographed assembly pathway

**DOI:** 10.1038/s41467-022-28828-1

**Published:** 2022-03-08

**Authors:** Khanh Vu Huu, Rene Zangl, Jan Hoffmann, Alicia Just, Nina Morgner

**Affiliations:** Institute of Physical and Theoretical Chemistry, Goethe University Frankfurt/Main, Max-von-Laue-Str. 9, 60438 Frankfurt/Main, Germany

**Keywords:** Mass spectrometry, Bioenergetics, Membrane proteins

## Abstract

F-type ATP synthases are multiprotein complexes composed of two separate coupled motors (F_1_ and F_O_) generating adenosine triphosphate (ATP) as the universal major energy source in a variety of relevant biological processes in mitochondria, bacteria and chloroplasts. While the structure of many ATPases is solved today, the precise assembly pathway of F_1_F_O_-ATP synthases is still largely unclear. Here, we probe the assembly of the F_1_ complex from *Acetobacterium woodii*. Using laser induced liquid bead ion desorption (LILBID) mass spectrometry, we study the self-assembly of purified F_1_ subunits in different environments under non-denaturing conditions. We report assembly requirements and identify important assembly intermediates in vitro and *in cellula*. Our data provide evidence that nucleotide binding is crucial for in vitro F_1_ assembly, whereas ATP hydrolysis appears to be less critical. We correlate our results with activity measurements and propose a model for the assembly pathway of a functional F_1_ complex.

## Abstract (web summary)

ATPases are the macromolecular machines for cellular energy production. Here the authors investigate factors that govern the assembly of the F_1_ complex from a bacterial F-type ATPase and relate differences in activity of complexes assembled in cells and in vitro to structural changes.

## Introduction

**Fig. 1:**
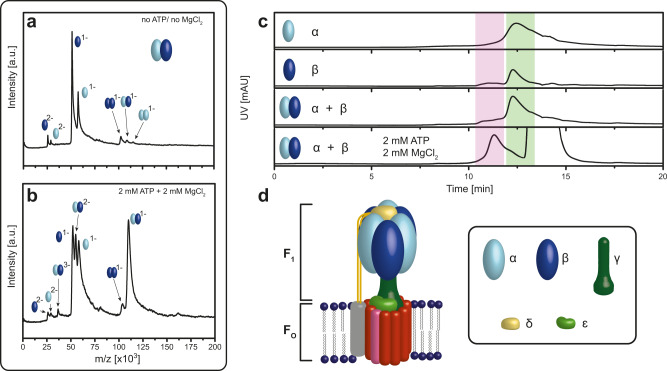
Dependence of ATP/Mg^2+^ in the assembly process of bacterial F-type ATP synthases. **a**, **b** LILBID spectrum of in vitro α and β assembly: the α- (light blue) and β-subunit (dark blue) either without **a** or with **b** 2 mM ATP and 2 mM MgCl_2_. In presence of ATP/Mg^2+^ the mass spectrum shows the formation of a specific αβ heterodimer assembly, but no higher αβ oligomers. **c** High-performance Liquid Chromatography (HPLC) of in vitro α (light blue) and β (dark blue) assembly: the addition of 2 mM ATP and 2 mM MgCl_2_ to α and β lead to αβ heterodimer formation displayed by a shift from 12.27 min (light green area) to ~11.26 min (light red area). The elution peak at 13.25 min is owing to the high ATP concentration. **d** Model of bacterial F-type-ATPase: the soluble module F_1_ is organized by α- (light blue), β- (dark blue), γ- (dark green), δ- (yellow) and ε-subunit (light green). Source data underlying **a**–**c** are provided as a Source Data file.

Life needs energy for numerous biochemical energy-consuming processes to maintain cellular functions, such as biosynthesis, membrane transport, regulatory networks, and nerve conduction. The energy is provided in the form of ATP, which cells have to convert using external energy sources such as light or nutrients. ATP synthases, the molecular machines employed for such ATP production are omnipresent and found in many biological systems, such as the inner membrane of mitochondria, the thylakoid membrane of chloroplasts, and the bacterial plasma membrane and therefore of high importance. Recently, the F-type ATP synthase has been highlighted as a novel drug target against *Mycobacterium tuberculosis*^1,2^ and the enzyme is gaining more and more interest as an attractive molecular target for the therapy of a variety of diseases (e.g., neuropathy, ataxia, retinitis pigmentosa syndrome, the familial bilateral striatal necrosis, one form of Leigh syndrome and Alzheimer’s disease)^3–5^. Therefore, the development of methods that allow further investigation of these complex macromolecular machines, the subunits’ assembly, and functionally relevant interplay, are of high interest. ATP synthases consist of a hydrophilic chemical-driven F_1_- and the ion-driven membrane-embedded F_O_-module, as shown in Fig. 1d. ATP is synthesized from adenosine diphosphate (ADP) and inorganic phosphate (Pi) in the six nucleotide-binding sites situated in the interface of the α and β subunits in the soluble hexameric F_1_ head. The energy is provided via a rotary mechanism of the membrane-bound F_O_ part driven by the electrochemical gradient across the membrane via channels at the interface of the peripheral stator and the c-ring, causing the c-ring to rotate^6–8^. Working in the reverse direction the ATPase/synthase can also hydrolyze ATP and function as an ion pump, dependent on the physiological demand of the living cell^7,9,10^.

The ATPase F_1_ part consists of nine subunits arranged as δ atop the hexameric head α_3_β_3_, which sits on a central stalk γε, whereas the membrane integrated F_O_ part comprises the peripheral stator unit ab_2_ and a membrane-embedded c-ring (Fig. 1). The c-ring is known to vary between different organisms, so far c-subunit stoichiometries between 8 and 17 have been reported^11–17^. The central stalk γε of F_1_ interacts with the c-ring as well as the hexameric head. The torque of γ leads to conformational changes in the β-subunits causing different nucleotide-binding affinities (termed open, loose, closed, and tight conformation) resulting in either ATP synthesis or hydrolysis^18,19^. For *Escherichia coli* the subunit ε was found to be critically important for the integration of the soluble part F_1_ with the membrane part F_O_^20–22^. It is as well known to exert a regulatory effect on bacterial ATPase activity^23–25^.

Furthermore, several studies focused on the assembly process of bacterial F-type ATPases. For instance, the sodium-driven Na^+^-F_1_F_O_-ATPase of *Acetobacterium woodii* has an unusual c-ring, the assembly of which has been shown to require the assembly factor AtpI, which could not be replaced by analog *Escherichia coli* AtpI^26^. This c-ring is comprised of two different types of subunits with a stoichiometry of 9:1 (c_2/3_:c_1_) as previously determined by laser-induced liquid bead ion desorption mass spectrometry (LILBID-MS)^27^. LILBID allows for the soft ionization of proteins and complexes and is therefore well suited to analyze stoichiometries and binding events. In the LILBID process, the aqueous sample of interest is introduced into the mass spectrometer via a piezo-driven droplet generator, producing droplets with a diameter of 50 μm at a frequency of 10 Hz. These are then irradiated with an IR laser tuned to the O-H stretching vibrations of water, leading to an explosive expansion of the droplets and setting the solvated biomolecule to be analyzed in a time-of-flight mass spectrometer.

Additional studies have shown the assembly of bacterial F_O_ to depend on the signal reconstitution particle pathway, SecYEG translocon, and YidC, which are required for the co-translational insertion of subunits a and b^28^. Although c-subunits and subunit b can be co-translationally reconstituted in the membrane independently of subunit a, the stable insertion of subunit a requires the presence of subunits b and c^29^.

The bacterial F_1_ module α_3_β_3_γε assembles independently of the membrane-bound F_O_ part^7,30^. In vitro reconstitution of isolated α-, β-, and γ-subunits support the possibility of in vitro formation of a functional active α_3_β_3_γ-subcomplex^31,32^. However, detailed studies of the assembly of bacterial F_1_ from single subunits are still missing. As two F_1_-specific chaperones, Atp11p and Atp12p are known to be functionally relevant for the assembly and solubility of β and α^33^ in mitochondria, whereas no such chaperones are identified in bacteria, the question arises if chaperones are relevant for the bacterial F_1_ assembly.

In this work, we use LILBID-MS to investigate the assembly process of the F_1_ complex of an *A. woodii* F-type ATPase. We probe the in vitro assembly of the separately expressed subunits, and propose a well-choreographed assembly pathway into functional F_1_ complexes. By comparing *in cellula* and in vitro (sub)complexes with regard to stoichiometries, ATPase activity, and stability, we conclude that—while chaperones may not be required for the correct assembly of these complexes—they assist in structurally fine-tuning the protein assembly, which is essential to reach full activity.

## Results

### Role of ATP for the in vitro assembly process of bacterial F-type ATP synthases

As the assembly mechanism of the bacterial F-type ATP synthase, in particular, for the soluble F_1_ module, is mostly still unclear, we aim to shed light on this process. We determine individual subunit interactions, relevant conditions and also investigate whether the assembly of all different subunits follows a specific order.

In a first step, we purified recombinant subunits (α, β, δ, and γε) of the soluble part F_1_ individually. Attempts to express the γ-subunit in *E. coli* separately yielded insoluble aggregates (inclusion bodies). Therefore, we cloned the genes *atpG* and *atpC* bicistronically in the expression vector and purified the central stalk γε to generate higher complex stability and protein solubility (Supplementary Fig. 8). To investigate individual assembly steps we incubated different subunit combinations under different assembly conditions and determined with LILBID-MS and High-performance liquid chromatography (HPLC) experiments, which complexes did or did not form. The resulting (sub)complexes represent key steps in the assembly process. To ensure the relevance of the observed in vitro subcomplexes for the *in cellula* processes in the cell and for comparison of structure, complex stability, and bioactivity, we then set out to produce homologous subcomplexes *in cellula*.

Incubation of recombinant α and β in vitro in ammonium acetate buffer without any additives shows no specific α/β-oligomerization into higher subcomplexes (Fig. 1a), suggesting that our experiment is missing something essential. The cytoplasmic concentrations of ATP in active cells are known to be approximately in the range of 2–5 mM^34^, which prompted us to test the relevance of ATP for this assembly. As MS resolution can be affected by additives, we stayed in the lower range of the native concentration and incubated the same proteins with 2 mM ATP and MgCl_2_ (Fig. 1). (Effect of ATP/Mg^2+^ on LILBID-MS spectra is shown in Supplementary Fig. 1). Figure 1b demonstrates that the addition of ATP/Mg^2+^ leads to specific αβ heterodimer formation only (no unspecific homodimers) with a charge state distribution (−1 to −3). Interestingly, no higher α/β subspecies can be identified, even though ATPases are known to form a hexameric head, containing three α- and β-subunits, respectively. As *in cellula* comparison, we purified and isolated an αβ complex from *E. coli*, which we then analyzed under the same in vitro conditions. The *in cellula* αβ complex showed the same heterodimer and no higher oligomeric states either (Supplementary Fig. 2). It should be noted that the in vitro formed αβ heterodimers need the presence of ATP/Mg^2+^ not only for formation but in order to remain stable, as can be seen from measurements, for which the buffer is slowly diluted during the LILBID-MS run (Supplementary Fig. 1). Reduction of the ATP/Mg^2+^ concentration during LILBID-MS goes along with an increase in the signal resolution but reduced complex stability. Interestingly the *in cellula* αβ complex seem less prone to dissociation at reduced ATP/Mg^2+^ concentration (Supplementary Fig. 2), which is the first hint that assembly of α and β in vitro and *in cellula* might lead to slightly different heterodimers.

For comparison with the LILBID-MS results we investigated the in vitro assembly of subunit α and β by an alternative analytical method: size exclusion chromatography (SEC) by HPLC, using the SEC column with a protein separation range of 10–700 kDa (Fig. 1c). The chromatograms show a shift into a shorter elution peak time confirming αβ heterodimer formation only upon the addition of 2 mM ATP and MgCl_2_. LILBID and SEC experiments both showed (Fig. 1a–c) that the additives ATP/Mg^2+^ are crucial for the in vitro assembly of subunits α and β of Na^+^-F_1_F_O_- ATP synthases of *A. woodii*.

### Assembly studies of single isolated subunits with next neighbors

**Fig. 2:**
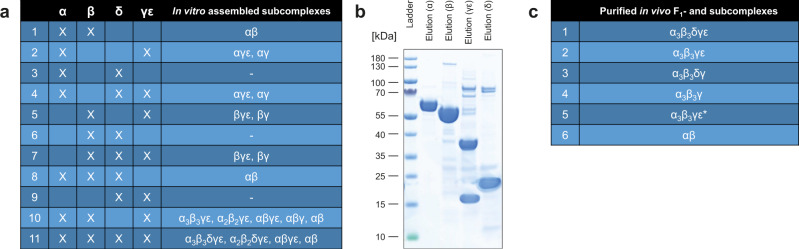
Summary of all subcomplexes obtained in vitro and *in cellula*. **a** List of all subcomplexes observed with LILBID-MS after in vitro assembly of isolated subunits in the presence of 2 mM ATP and 2 mM MgCl_2_. **b** SDS-PAGE (NuPAGE 4–12% Bis-TRIS) of single purified subunits α, β, γε, and δ. Affinity purification of α with Strep-Tactin- and β, γε, and δ with Ni-chelating chromatography. *N* = 3 independent experiments. **c** List of in *E. coli* heterologously purified F_1_ and subcomplexes. All complexes were analyzed with LILBID-MS and SDS-PAGE (Supplementary Fig. 7). Source data underlying Fig. 2a are provided as a Source Data file.

After determination of the appropriate assembly conditions, we are now in a position to analyze the entire assembly process including subunits α, β, δ, and γε. As it was not yet clear, if the assembly follows a clear step by step order we tested every possible combination of subunits for binding in the same manner as described above and investigated which prior binding steps are prerequisite for other binding steps to follow (all binding experiments are listed in Fig. 2a). Figure 2 gives an overview of all possible subcomplexes which we observed after incubation of different combinations of subunits. This provides information about next neighbor interactions and the order of assembly steps within the F_1_ complex. As mentioned above we can observe αβ heterodimer assembly under the right assembly conditions—but no higher-order oligomers, if just these two subunits are present (Fig. 1b). Similarly, we observe that upon incubating either subunit α or β with the γε complex, a single subunit α or β can bind to the γε complex, forming αγε or βγε complexes, respectively. These complexes are seen under soft laser conditions (laser energy 8 mJ). Already medium laser desorption conditions, allow the ε-subunit to dissociate from the γε complex (Supplementary Fig. 9c, d), giving rise to αγ or βγ complexes. This shows that α or β bind to the γ-subunit, whereas the ε-subunit is only weakly bound to γ and not directly involved in the interaction with α or β.

**Fig. 3:**
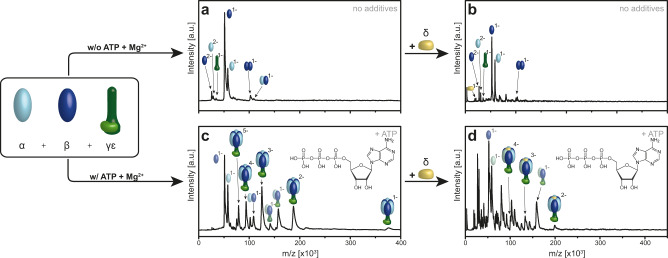
ATP/Mg^2+^ dependence of the in vitro assembly process of the full F_1_ complex of Na^+^-F_1_F_O_- ATP synthases of *A. woodii*. The α- (light blue), β-subunit (dark blue), γε-complex (dark and light green) were incubated at 4 °C for 1 h either without (**a**) or with (**c**) 2 mM ATP and 2 mM MgCl_2_. Then the δ-subunit (yellow) was added (**b**, **d**). **a**, **b** No specific assemblies into higher F_1_-subcomplexes could be detected without ATP/Mg^2+^. The appearance of the γ subunit alone is due to dissociation. (See as well Supplementary Fig. 8c). **c**, **d** In presence of ATP/Mg^2+^ the fully assembled F_1_ can be identified with additional F_1_ subcomplexes (e.g., α_2_β_2_γε, αβγε, αβγ) implying pairwise specific αβ heterodimer association. The δ-subunit can only bind when a complex containing the hexameric head domain α_3_β_3_ is already preformed. Source data are provided as a Source Data file.

As subunit α and β alone can only form heterodimers and only a single α or β can bind to γ if incubated alone, the next step was to purify and incubate recombinant α, β, and γε in presence of 2 mM ATP/MgCl_2_. Mass spectrometric analysis showed the formation of the α_3_β_3_γε-subcomplex which we could identify at a mass of 378 ± 13 kDa (theoretical protein mass 378.272 kDa) and a charge state distribution from −1 to −5 (Fig. 3). In contrast, no higher subcomplex formation was observed if the same experiment was performed without ATP/Mg^2+^. This implies that these additives are vital for the entire in vitro reconstitution process providing complex stability.

Including the δ subunit in the incubation then led to its association with the preformed α_3_β_3_γε complex resulting in the fully assembled F_1_-complex α_3_β_3_δγε with a determined protein mass of 399 ± 8 kDa (theoretical mass: 399.809 kDa) and a charge state distribution of −1 to −3 (Fig. 3d). The spectrum shows the fully assembled F_1_-complex, as well as subcomplexes α_2_β_2_γε, αβγε, and αβγ, which could stem from incomplete assembly or dissociation. Surprisingly our spectra show no subcomplexes with an odd number of α or β subunits, indicating that the αβ heterodimer is the stable basic binding block from which the hexamer is built up. No δ is seen binding to any of the single subunits, which is in line with studies showing the interaction of δ with α-subunits only if complexed with other F_1_-subunits^35^. No δ binding is seen for any of the mentioned subcomplexes either (Fig. 2a and Supplementary Fig. 9a, b), suggesting the fully assembled αβ-hexamer to be a prerequisite for binding of δ. Combined the observed on-pathway subcomplexes en-route to the F_1_ complexes allow proposing the ATPase assembly to occur only via the binding of preformed αβ heterodimers onto the γ-subunit, to form the hexameric head, which then allows for binding of δ. As γ could not be purified separately we could not perform binding experiments with and without ε to determine, if ε is a prerequisite for binding of the αβ dimers onto γ, or if the hexameric head might form around γ independently of ε.

For comparison, we then attempted to purify and isolate all proposed on-pathway subcomplexes *in cellula*. All expected complexes (Fig. 2c) could be purified. In addition, we could purify complexes lacking ε, but still include the hexameric head. This allowed us to conclude that the hexameric head forms around γ independently of ε, which therefore has multiple entry points into the assembly pathway. Mass spectrometric analysis (Supplementary Fig. 7) reveals, as for the in vitro complexes, only complexes with the same amount of α and β subunits, supporting the αβ heterodimer as a basic building block. Incubation of δ with the *in cellula* complex as well confirms our in vitro results, which showed δ to only bind onto the complete αβ hexamer.

Interestingly, the *in cellula* α_3_β_3_γε complex (Supplementary Fig. 7) shows a significantly higher complex stability even without the addition of ATP/Mg^2+^ than the in vitro complex (Supplementary Fig. 2e, f). This difference could be very interesting, as it might show that there are deviations in the *in cellula* and in vitro assembly, possibly owing to structural differences of the proteins. An alternative explanation would be steric hindrances due to the tags, which are part of our in vitro purified proteins. The in vitro studies were performed on N-terminal StrepI-α and His_6_-β, C-terminal His_6_-δ, and γε (His_6_-tag located on subunit ε), whereas the *in cellula* complex was expressed with only one N-terminal His_6_-tag on the β-subunit. For comparison, we purified a complex, which includes the same tags on every subunit as our isolated purified subunits, which we name α_3_β_3_γε* for distinction. The mass spectrum in Supplementary Fig. 6a shows that the tags are not responsible for destabilization of the complex.

Remarkably the *in cellula* complexes are not only stable without ATP/Mg^2+^ addition that is crucial for the in vitro complexes, but even the last assembly step association of δ to *in cellula* purified α_3_β_3_γε and α_3_β_3_γε* pre-complexes forming the full F_1_-complex (Supplementary Fig. 6) is ATP/Mg^2+^ independent.

### The role of ATP hydrolysis *vs*. nucleotide binding for the in vitro assembly process of bacterial F_1_-ATPase

The important role of ATP hydrolysis for the function of ATP synthases is unquestioned and we have as well observed the significance of ATP for complex assembly. Nevertheless, it is not yet clear, whether ATP hydrolysis or only nucleotide binding is required for the F_1_ assembly process. Therefore, we repeat the described assembly experiments with α, β, and γε, but replace ATP by non-hydrolysable ATP analogs, such as ADP, adenylyl-imidodiphosphate (AMP-PNP) and adenosine 5′-[γ-thio]triphosphate with a concentration of ATP/ATP analogs and Mg^2+^ at a concentration of 2 mM.

**Fig. 4:**
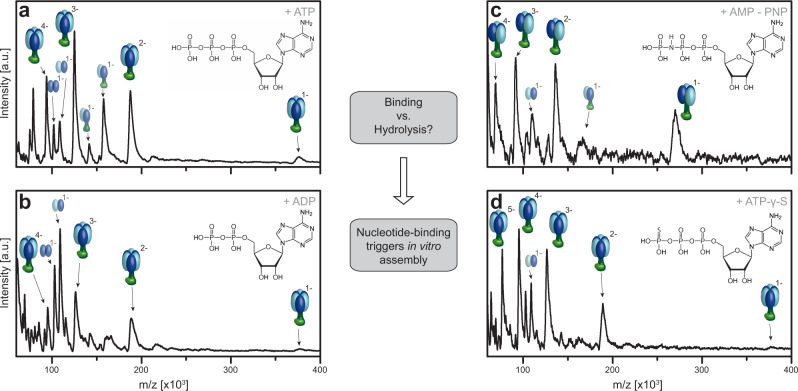
Role of ATP hydrolysis *vs*. nucleotide-binding in the assembly process to F_1_ of bacterial F-type ATP synthases. LILBID spectra of in vitro α, β, γε assembly: The α- (light blue), β-subunit (dark blue), γε-complex (dark and light green) were incubated with 2 mM MgCl_2_ and 2 mM of a nucleotide at 4 °C for 1 h. Complex formation is observed after incubation with ATP (**a**), AMP-PNP (**b**), ADP (**c**), and ATP-γ-S (**d**). Full α_3_β_3_γε assembly can be identified next to small amounts of other F_1_ subcomplexes (e.g., α_2_β_2_δγε α_2_β_2_γε, αβγε, αβγ), for ATP as well as the non-hydrolysable ATP analogs, apart from AMP-PMP, where the highest observed complex is α_2_β_2_γε. Source data are provided as a Source Data file.

The obtained MS spectra with non-hydrolysable ATP analogs (Fig. 4b–d) shows the successful formation of larger F_1_-subcomplexes. Interestingly all ATP analogs allow the formation of α_3_β_3_γε apart from AMP-PNP, which generates only α_2_β_2_γε subcomplexes and not the expected α_3_β_3_γε complex; which could be due to differences in the chemical geometry of AMP-PNP. We speculate that the free electron pair of nitrogen might sterically inhibit the assembly of the last αβ heterodimer to the complete α_3_β_3_ hexamer. Generally, this confirms that ATP hydrolysis is not required for assembly, but nucleotide binding is the essential factor triggering the in vitro complex formation.

### Importance of charged residues in the catalytic sites of bacterial F-type ATP synthases

**Fig. 5:**
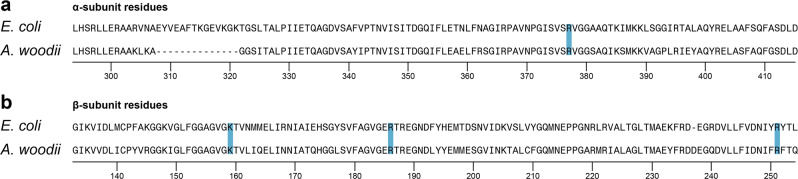
Amino-acid sequence alignment of α- and β-subunit residues for *E. coli* and *A. woodii*. **a** α-subunit residues. **b** β-subunit residues. Positions of positively charged amino acids that were mutated are marked in blue.

After determining the crucial effect of ATP binding for ATPase assembly, we want to shed more light on this process. Recent studies have shown the importance of charged residues for the ability of _Pi_-binding at the catalytic sites of the αβ interface of the hexameric head. The replacement of charged amino acids with neutral residues induced reduced cell growth and loss of ATPase activity, suggesting the electrostatic interactions between amino acids to be crucial for initial phosphate binding in the catalytic sites^34^. Specifically, four residues β[K155], β[R182], β[R246], and α[R376] were shown to be critical^36–38^. We want to deduce if these mutations affect ATPase activity via their influence on the ability of the complex to bind/hydrolyze ATP or if the effect might be even more drastic and influence the complexes assembly. Therefore, we introduced homologous point mutations in conserved regions in α[R363Q, R363K] and β[K159Q, R186Q, R251Q] in the *A. woodii* operon (Fig. 5).

**Fig. 6:**
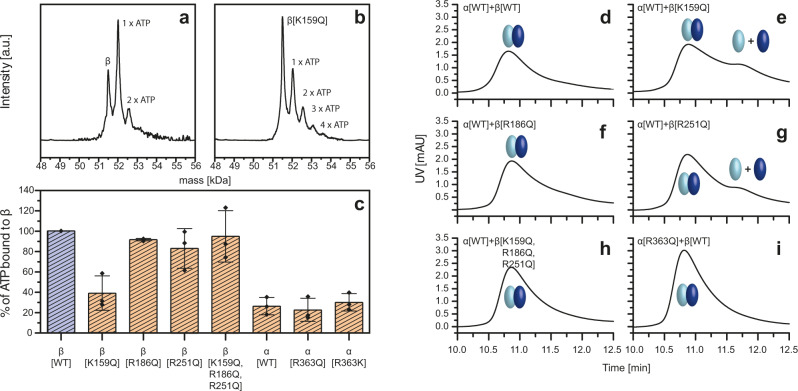
Dependence of ATP-binding of α, β and their mutants and the formation of αβ heterodimers. **a**, **b** ATP-binding: deconvoluted nESI-MS spectra of ATP-binding of β[WT] (**a**) and β[K159Q] (**b**). Corresponding spectra for all α and β mutants are shown in Supplementary Fig. 4. **c** Summary of ATP-binding of β[K159Q], β[R186Q], β[R251Q], β[K159Q, R186Q, R251Q], α[WT], α[R363Q], and α[R363K] relative to β[WT]. Data are presented as mean values with error bars showing the SD, with individual data superimposed. **d**–**h** HPLC measurements: in vitro αβ heterodimer assembly of α[WT] (light blue) with β[WT] (dark blue) and β mutants. **i** HPLC measurement: In vitro αβ heterodimer assembly of β[WT] (dark blue) with α[R363Q] (light blue). Data were collected in biological replicates (*n* = 3). Source data are provided as a Source Data file.

To investigate the effect of these charged residues in the catalytic site we first analyze the binding efficiency of ATP to α, β, and different mutants with nano electrospray-ionization (nESI) (Fig. 6), which allows observing the number of bound ATP. The deconvoluted MS-spectrum in Fig. 6a shows three peaks corresponding to β[WT] with zero, one, or two bound ATPs. The dominating species is clearly the one with one bound ATP, indicating specific binding. In contrast, peaks trailing for the mutant β[K159Q] (Fig. 6b) suggest non-specific ATP binding only. Figure 6c shows the differences in ATP binding for the investigated α and β subunits and their respective mutants. The β[K159Q] mutant demonstrates a decrease of ATP binding ~60%, if compared with β[WT]. A less pronounced effect is seen for β[R251Q]. No significant differences in ATP binding were observed for α[WT] and its mutants that have a generally lower ATP-binding efficiency compared to β[WT].

Seeing that charged residues in the catalytic site can influence ATP-binding, we wanted to assess, if reduced ATP-binding affinity had an effect on the in the vitro assembly process. Therefore, we performed the same HPLC and MS experiments as for the wildtype α-β incubations. Figure 6d–h shows with HPLC the formation of αβ heterodimers at an elution peak time of 10.8 min for α[WT] with each β construct. Similarly, the in vitro assembly of β[WT] with α[R363Q] (Fig. 6) or α[R363K] (Supplementary Fig. 3a) shows heterodimer formation. Interestingly, the HPLC runs for α[WT] with β[K159Q] or β[R251Q] (Fig. 6e/g) the mutants, which reduced ATP binding, show additional peaks at ~11.7 min, suggesting the presence of unassembled monomers and therewith less-efficient binding.

**Fig. 7:**
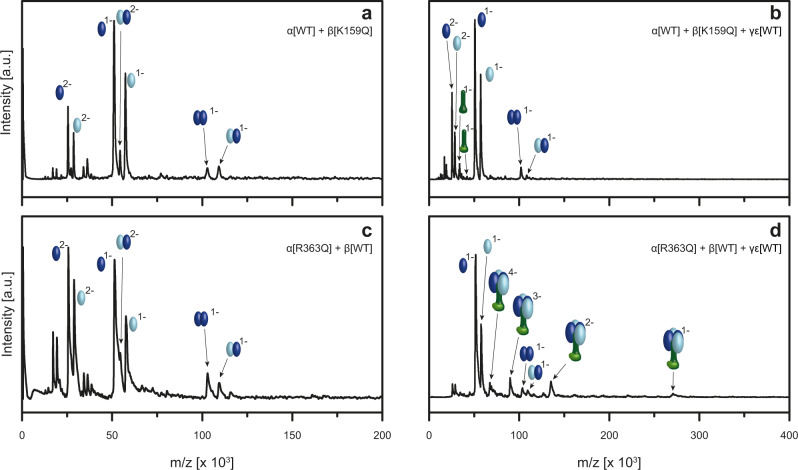
Role of charged residues in the catalytic site for the assembly process analyzed by LILBID-MS. **a** In vitro assembly of α[WT] (light blue) with β[K159Q] mutant (dark blue) shows no significant formation of the αβ heterodimer, which we observe for the α[WT] and β[WT] (Fig. 1b). **b** The addition of the central stalk γε (dark and light green) does not lead to the formation of the desired α_3_β_3_γε complex. **c** In vitro assembled α[R363K] (light blue) and β[WT] (dark blue) revealed reduced αβ heterodimer assembly. **d** The biggest complex that is observed after addition of γε (dark and light green) is an α_2_β_2_γε subcomplex with charge states (−1 to −4). Source data are provided as a Source Data file.

In vitro assembly of the α and β mutant constructs was then probed by LILBID-MS. Interestingly, we observe that under the same experimental conditions, which show stable heterodimers of wildtype α and β (Fig. 1b), we do not detect stable αβ heterodimers for α[WT] incubated with β[K159Q] (Fig. 7a) or any of the β mutants (Supplementary Fig. 5). This suggests that all β mutations affect stability to the extent that dimers can still be observed with HPLC (to a different degree) but are much reduced in the LILBID spectra. Similarly, we see clearly decreased dimerization for β[WT] with α[R363Q] (Fig. 7c). A point mutation at the same position which retains the charge of the original amino acid (α[R363K]) shows no effect, as it does not hinder the formation of the αβ heterodimer (Supplementary Fig. 3).

As our experiments so far indicate that the formation of the αβ heterodimer is decisive for further complex formation, the mutations, which impede the heterodimer, should affect the formation of higher-order F_1_-complexes as well. Our mass spectra confirm that incubation of α[WT], β[K159Q] and the central stalk γε does not lead to the α_3_β_3_γε complex (Fig. 7b), which we observed with the wildtype β (Fig. 3c). The same result is obtained with all other β mutants, β[R186Q], β[R251Q] and β[K159Q, R186Q, R251Q], highlighting the important role of the charged residues in subunit β not only for the catalytic activity but as well for the F_1_ assembly (Supplementary Fig. 5). In contrast assembly of the mutant α[R363Q], β[WT] and the central stalk γε generates a larger complex—but interestingly not the expected hexameric head but subcomplex α_2_β_2_γε (Fig. 7d). This indicates that replacing the arginine with the uncharged glutamine in subunit α has an effect on the assembly process, which is less pronounced than the effect of the above-mentioned substitutions in subunit β, but still hinders the formation of the correct αβ hexamer.

### Order of assembly steps into F_1_

**Fig. 8:**
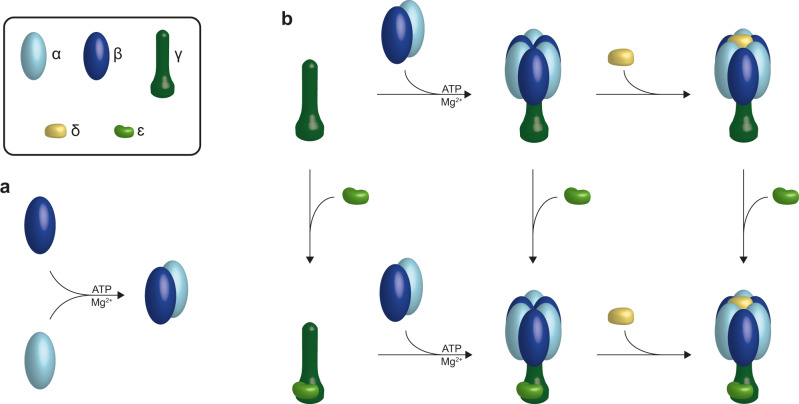
Order of assembly steps into bacterial F_1_. **a** As a first step α-subunits (light blue) and β-subunits (dark blue) form αβ heterodimers, which requires the presence of nucleotides and Mg^2+^. **b** As next step three of these heterodimer building blocks assemble pairwise to the central stalk γ (dark green). Only after the formation of the full hexameric head, the δ-subunit (yellow) is able to bind. The ε-subunit (light green) can join into the assembly process at all intermediate step en-route to the F_1_ complex.

The combined results obtained with LILBID and nESI-MS and HPLC allow us to establish all subcomplexes which form in vitro en route to the full complex as well as the necessary assembly conditions. As these assemblies take place in a minimal environment, compared to a cell, we need to guarantee that the observed subcomplexes are not just the result of aggregation, but can occur in the same manner in the cell. Therefore, we expressed the subunit combinations, which we found in our assembly experiments directly in *E. coli* cells for comparison. We were able to obtain all anticipated subcomplexes with the same stoichiometries (Supplementary Fig. 7). Based on these findings, we can follow the individual assembly steps and establish their order for the formation of bacterial soluble F_1_ domain of F-type ATP synthases (Fig. 8).

Overall our results show that, α- and β-subunits assemble specifically to αβ heterodimers only in presence of nucleotides and Mg^2+^ (Fig. 1, Fig. 3 and Fig. 4) while surprisingly not forming higher subcomplexes (e.g., αβ_2,_ α_2_β, α_2_β_2_ etc.) least of all the expected hexameric head (α_3_β_3_). The α and β subunits can both bind individually to the central stalk γ, but only little binding is observed and in a maximal copy number of one (Supplementary Fig. 9c, d), unless α and β dimerize first. Three pairs of αβ heterodimers bind either to the central stalk γ or a preformed γε complex to generate stable α_3_β_3_γ or α_3_β_3_γε complexes, respectively. The relevance of these in vitro results is shown in our heterologously expressed subcomplexes α_3_β_3_γ and α_3_β_3_γε which we can isolate as stable and bioactive complexes *in cellula* (Supplementary Fig. 7). The δ-subunit binds to the *in cellula* or in vitro pre-complexes containing the hexameric head (Fig. 3d and Supplementary Fig. 6), which generates the full F_1_ complex.

As isolated subunit γ could not be purified in *E. coli* for stability reasons, we purified γε (Supplementary Fig. 8), so our assembly studies did not include binding of ε and γ analogously to all the other binding experiments. The complete aggregation of the γ-subunit into inclusion bodies in the bacterial matrix during the overexpression could suggest that the ε-subunit is essential for the stabilization of the central stalk (Supplementary Fig. 8), which would place binding of ε to γ as a first step parallel to the formation of the αβ heterodimer, before further assembly steps occur. Nevertheless, expression of *in cellula* subcomplexes showed that α_3_β_3_γ can be expressed as a stable subcomplex, which indicates that binding of ε is not an essential prerequisite for any further assembly to the F_1_ complex. ε could therefore enter the assembly network at different times, as we indicate in Fig. 8.

Interestingly, we observe some differences between our *in cellula* and in vitro complexes: the *in cellula* preformed complexes are stable in a buffer without nucleotide- and Mg^2+^ and the binding of δ to any complexes including the hexameric head can then be performed in vitro without nucleotide- and Mg^2+^ addition. The same assembly experiment with the in vitro formed α_3_β_3_γε complex requires nucleotide- and Mg^2+^ addition, to hinder disaggregation of the complexes. This could be explained, if the F_1_-subcomplexes formed *in cellula* and in vitro shared the same assembly pathway and stoichiometry, but a structural change occurring in the cell to stabilize the formed complex, potentially by more tightly retaining bound nucleotides, were missing in vitro, which is a question we will come back to later. Figure 8 depicts a full summary of the different assembly steps of the soluble part F_1_ as determined by our experimental data. The entire assembly process follows a choreographed pathway, which allows for efficient construction of the F_1_ complex.

### ATPase activity of in vitro assembled *A. woodii* αβ and α_3_β_3_γε complex

In general, many macromolecular complexes need molecular chaperones, which assist for example in the conformational folding or assembly processes. In mitochondria, it is known that the F_1_- assembly of the hexamer of alternating α- and β-subunits requires two specific chaperones, Atp11p and Atp12p, that bind transiently to β and α^33,39^. Our previous results demonstrate that the bacterial F_1_- assembly can occur independently of chaperones. This was a surprising finding, as a generally accepted hypothesis expects the bacterial F_1_- assembly to as well depend on chaperones. So far one such Atp12p homolog has been identified in proteobacteria^40^. The question arises, if chaperones are not needed at all, or if their presence might be required, if not for assembly, then to guarantee correct function and activity.

**Fig. 9:**
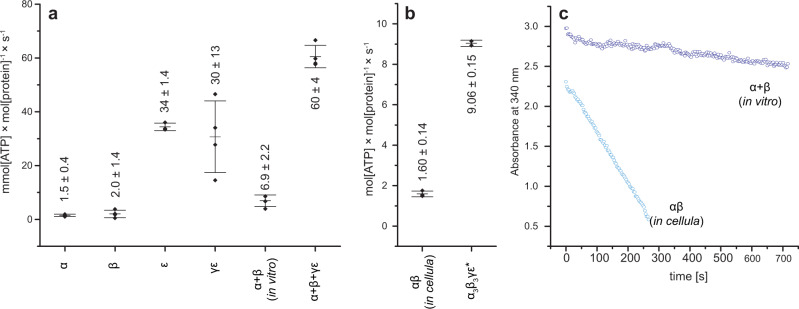
ATP hydrolysis of isolated subunits and in vitro and *in cellula* assembled complexes. **a** Isolated α, β, ε, γε and in vitro assembled αβ and α_3_β_3_γε complexes. **b** *In cellula* αβ and α_3_β_3_γε^*^ complexes. Data are presented as mean values with error bars showing the SD, with individual data superimposed. **c** Spectroscopic comparison of the decrease in absorbance at 340 nm per time [s] in the enzyme-coupled ATPase activity assay of the in vitro assembled αβ from isolated α and β (5.5 µM each) relative to the *in cellula* produced αβ (0.5 µM protein complex) in 100 µL (100 mM TRIS, 100 mM maleic acid, 5 mM MgCl_2_, 3 mM phosphoenolpyruvate (PEP), 4 mM ATP, 0.5 mM NADH, 10 units L-lactate dehydrogenase (L-LDH), 10 units pyruvate kinase (PK), pH 7.5). In vitro data were collected in biological quadruplicates (*n* = 4) except *ε* with three biological replicates (*n* = 3) and *in cellula* data were collected in biological triplicates for αβ (*n* = 3) and technical duplicates for α_3_β_3_γε* (*n* = 2). Source data underlying Fig. 9a and b are provided as a Source Data file.

To answer this question, we investigated our in vitro assembled complexes for bioactivity. We performed ATP hydrolysis experiments with all of the separately expressed constructs (α, β, γε, and ε) and the in vitro assembled αβ and α_3_β_3_γε complexes using an enzyme-coupled activity assay^41,42^. For in vitro complex formation, the subunits were incubated with 4 mM MgCl_2_ and ATP with subunit stoichiometries of 1:1 for the αβ heterodimer, and 3:3:1 for α_3_β_3_γε. All subunits and subcomplexes showed moderate ATPase activity (Fig. 9a).

We could determine ATPase activities for the β- and α-subunit of 1.5 ± 0.4 and 2.0 ± 1.4 mmol [ATP]/(mol[protein] · s), respectively. These are quite similar and do not reflect the threefold higher ATPase activity of α previously seen in *E. coli*^32^.

As isolated subunit ε was already known to bind ATP^43^ and has to undergo large conformational changes to regulate the ATPase^44^ we hypothesized that ε as well undergoes hydrolysis. Interestingly, the isolated ε-subunit showed a hydrolytic activity of 34 ± 14 mmol [ATP]/(mol[protein] · s), which is one magnitude higher than that of the single catalytic β-subunit. The enzymatic hydrolytic activity of γε was measured at 30 ± 13 mmol [ATP]/(mol[protein] · s) placing the overall activity of γε very similar to ε, suggesting no contribution from γ.

The ATPase activity calculated for the in vitro assembled αβ heterodimer is 6.9 ± 2.2 mmol [ATP]/(mol[protein] · s), assuming incubation leads to full complex formation. As this might not be the case, all molar activities of in vitro complexes have to be seen as a conservative estimate—if full complexation could be guaranteed the observed values would likely be higher. Nevertheless, the observed hydrolytic activity is about twice the sum of the individual α and β activities, supporting the relevance of the αβ heterodimer as a building block and working unit of the ATPase. Surprisingly this value stays clearly below the hydrolytic activity of ε.

The reconstitution based on the incubation of α, β, and γε resulted in a hydrolytic enzymatic activity of 60 ± 4 mmol [ATP]/(mol[protein] · s). Interestingly, the activity of this in vitro assembly is not much higher than the sum of the ATPase activities of the utilized subunits. The increased activity (factor 1.5) of the now complexed subunits stems mainly from the increase of activity seen for the formation of the three required αβ heterodimers.

Similarly, the *in cellula* purified αβ and α_3_β_3_γε* were investigated. The ATPase activity for both is noticeably beyond anything we observed for in vitro complexes (1.60 ± 0.14 and 9.06 ± 0.15 mol [ATP]/(mol[protein] · s), respectively) (Fig. 9a and b). These values cannot be compared directly with those obtained for in vitro complexes, for which the individual subunits do not fully form complexes. Nevertheless, the activity of the *in cellula* αβ heterodimer alone is already much higher than that of the in vitro assembled α_3_β_3_γε complex. Similarly, the *in cellula* α_3_β_3_γε^*^ shows an activity way beyond the sum of its components. This indicates that the in vitro conditions allow for the correct assembly of the functional subunits, which then retain their individual ATPase activity, while the cellular environment allows for modifications, which increase the ATPase activity of the αβ heterodimer and the whole ATPase, way beyond that of the sum of its parts.

### Stability studies of *in cellula* vs in vitro assembled heterodimers with ion mobility MS

The observed differences in bioactivities as well as stability without ATP/Mg^2+^ for our *in cellula* and in vitro complexes suggest that additional effects besides assembly are required to form an active complex. We assume conformational changes to play a role, possibly triggered by chaperones.

**Fig. 10:**
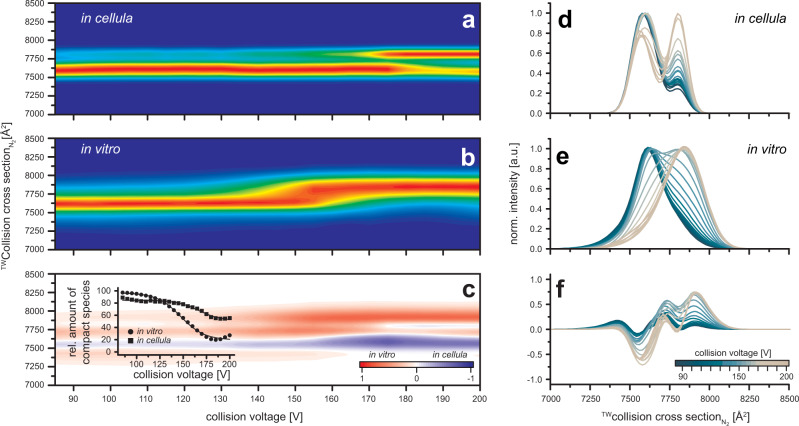
CIU fingerprints of the *in cellula* and in vitro assembled αβ heterodimer in the presence of ATP and Mg^2+^. **a** *In cellula* assembled αβ heterodimer. **b** In vitro assembled αβ heterodimer. Exemplary spectra are shown in Supplementary Fig. 10. At low collision voltages the IM signal of the αβ heterodimer appears at 7595 Å^2^ for the *in cellula* and at 7630 Å^2^ for the in vitro assembled αβ heterodimer, respectively. At higher collision voltages the signal of the in vitro assembled αβ heterodimer unfolds, resulting in a signal at 7851 Å^2^. At 152.6 V 50% has reached the unfolded state. In contrast, increasing the collision voltage in the case of the *in cellula* formed αβ heterodimer leads to a signal appearing at 7802 Å^2^. 50% unfolding is never reached—the maximal unfolding of 47% is observed at 200 V. **c** The difference plot of both fingerprints illustrates the change regarding unfolding behavior between in vitro and *in cellula* assembled heterodimers. **d**–**f** CCS distribution for different collision voltages for in vitro (**d**) and *in cellula* (**e**) assembled heterodimers, and the difference plot (**f**). The overall feature of the in vitro αβ is clearly broader than for the *in cellula* αβ.

The relevance of the αβ heterodimer formed as a first step in the ATPase assembly and the differences observed in our *in cellula* and in vitro studies, makes the heterodimer a very interesting candidate to investigate if the changes in activity can be correlated to structural changes. Noticeable structural rearrangements should be accompanied by a change in collision cross-section (CCS), which can be monitored by ion mobility (IM) MS, while changes to the intrinsic stability can be revealed by differences in collision-induced unfolding (CIU) experiments. To validate our assumption, we investigate the heterodimer for differences between *in cellula* and in vitro formed complex. We select in both cases the 22-times charged αβ heterodimer in our nESI mass spectra as precursor ion which is analyzed via IM MS in dependence of the applied collision voltage. IM allows the separation of ions based on their mobility through an inert gas under the influence of an electric field. Increasing the collision voltage can lead to CIU and then dissociation of the complex. The energy required to cause CIU can be correlated to the complex stability. In the resulting IM fingerprint of the *in cellula* assembled αβ heterodimer, the IM signal appears for low collision voltages at 7595 Å^2^ and 7630 Å^2^ for the *in cellula* and the in vitro complex, respectively (Fig. 10) These values correspond to the compact feature of the folded complexes and already suggest slight structural differences. An increase of collision voltage leads to protein unfolding, which is accompanied by an increase in CCS. The original IM signal decreases partially in favor of a signal at 7802 Å^2^ (*in cellula*) and 7851 Å^2^ (in vitro), representing the corresponding unfolded structure of the αβ heterodimer. 50% of the in vitro assembled αβ heterodimer unfolds at collision voltages of 152.6 V and appears at a slightly higher CCS compared to the *in cellula* assembled αβ, which preserves the compact state for >50% even at the highest collision voltages. This indicates that the in vitro structure is less stabilized against unfolding than the *in cellula* assembled αβ. This can be seen more clearly in the difference plot (in vitro minus *in cellula*, Fig. 10c), which helps to compare specific features of each fingerprint directly. In addition, it reveals a significantly broader IM signal for the compact, as well as the unfolded state for the in vitro assembled αβ heterodimer indicating a less homogenous structure for the in vitro than for the *in cellula* αβ heterodimer. We conclude that the more defined structure and increased stability of the *in cellula* heterodimer are structural properties, which can be correlated to the increased ATPase activity.

## Discussion

The aim of this study is to better understand the assembly process of an ATPase—a large heterogeneous macromolecular complex, with eight different subunits. We determined conditions required for the whole in vitro assembly process into the soluble part F_1_ of *A. woodii* ATPase from single purified F_1_-subunits. We followed the assembly process in vitro step by step from subunits via the subcomplexes, which form (or do not form) after incubation of different subunit combinations. To verify that the observed complexes on pathway to the F_1_ complexes are meaningful and can assemble in the same stoichiometries in the cell, we expressed a variety of *in cellula* F_1_ subcomplexes. We could purify all of the on-pathway complexes *in cellula*, which allowed comparison of in vitro *vs. in cellula* formed complexes.

Although the presence of nucleotides and Mg^2+^ is known to be required for F_1_ assembly of *E. coli*^31^, for the thermophilic bacterium PS3 the formation of an α_3_β_3_ hexamer has been shown to occur independently of ATP/Mg^2+^ ^45^, showing that this is not a universal requirement. Here we find for the mesophilic *A. woodii* that the presence of ATP/Mg^2+^ is essential for complex formation (Fig. 1, Fig. 3), up to the α_3_β_3_γε. We reveal that it is ATP binding that triggers the in vitro assembly and not ATP hydrolysis (Fig. 4).

We could demonstrate that the incubation of α and β leads to the formation of the heterodimer without the formation of any higher αβ oligomers. Only incubation in the presence of the central stalk γε allowed further assembly into larger complexes including the hexameric head-on the pathway to the F_1_ (Fig. 2). The lack of complexes with odd numbers of α and β subunits (Figs. 3, 4, Supplementary Fig. 7) shows that the formation takes place with the αβ heterodimers as basic building blocks. As we were able to express and purify intact α_3_β_3_γ complexes without the presence of ε we can conclude that it is the central stalk subunit γ, which is crucial for generating a stable α_3_β_3_ hexagon out of αβ heterodimers, while ε can join at different stages of the assembly. Including δ in our assembly studies could show that δ does not bind to isolated α or β (Supplementary Fig. 9a–b). Only after the formation of the hexameric head, the subunit δ can bind, resulting in the full F_1_ complex (Fig. 2, Supplementary Fig. 6), which can occur ATP/Mg^2+^ independent (Supplementary Fig. 6). Overall, our in vitro assembly experiments in combination with the complexes which can be assembled *in cellula* give a full picture of a well-choreographed assembly process, as shown in Fig. 8.

Point mutations in the catalytic interface of α (at position R363), and β[K155, R182, R246] which replace positively charged amino acids with neutral residues, demonstrated that such modifications can but do not have to have a direct effect on ATP binding efficiency. HPLC runs still showed the in vitro assembly of αβ heterodimers for all combinations of one wildtype and one mutated subunit (Fig. 6d–i), albeit with affected complex stability, as incomplete dimer assembly is observed for those mutants with reduced ability to bind ATP. This effect on stability is even more pronounced in the LILBID-MS results, which show reduced or no αβ dimer for all mutants which substitute a charged with a neutral amino acid (Fig. 7a and b, Supplementary Fig. 5). Substitution of one charged amino acid for another α[R363K] had no influence. This demonstrates that those point mutations, which remove a charge in the αβ interface, cause a decrease of αβ heterodimer stability. In vitro assembly experiments with α and any β mutant, which included γε did not yield any higher complexes. An interesting finding was that for the α mutant α[R363Q], stable αβ heterodimers and even higher F_1_ subcomplexes, including one or two heterodimers (Fig. 7c and Supplementary Fig. 3) can still form, albeit no complex with a complete hexameric head can be detected (Fig. 7d). A similar disturbance was found for incubations of wildtype subunits with AMP-PNP, which might sterically influence the assembly. This shows that even if αβ dimer formation is possible, the assembly of the hexameric head, which has to function via large conformational changes in a very tightly controlled manner, is a precise process, which can be hindered by small disruptions.

Our in vitro assembly studies prove that no direct involvement of chaperones is necessary for the correct reconstitution of the F_1_ complex from the individual subunits (Fig. 8). Based on the known relevance of chaperones for complex formation in cells this is a surprising finding. Therefore, the question arises: If chaperones are not essential for the in vitro assembly of the F_1_-ATPase, could they be relevant to ensure the bioactivity and function of the enzyme? To shed light on this question, we correlate our assembly results with an enzyme-coupled activity assay for single isolated F_1_-subunits, *in cellula* and in vitro formed complexes (Fig. 9). The first building block is the αβ heterodimer. The in vitro assembled αβ shows an activity, which is about twice as high as the mere sum of the α and β activity. This effect is not big, so it could be based on the ATP hydrolysis taking place in the αβ interface, which would offer a better binding pocket for the nucleotide. A much larger effect is seen in the comparison with the *in cellula* αβ dimer. Mass spectrometric analysis (Fig. 1b) and HPLC (Fig. 1c) measurements confirm the efficient, albeit not complete heterodimer assembly, so exact comparison of *in cellula* and in vitro activity is difficult. Assuming that about half of the possible dimers form in vitro, a conservative estimate would put the activity of the *in cellula* purified protein complex at least a factor of 100 above that of the *vitro* assembled complex. This suggests a principle difference in the heterodimers, leading to much higher bioactivity of complexes formed in a cell. This could be explained by a structural difference of the in vitro and the *in cellula* formed heterodimer.

To further investigate this assumption, we perform stability studies with *in cellula* (Fig. 10a, d) and in vitro (Fig. 10b, e), formed αβ heterodimers by means of mass spectrometry CIU experiments, which we monitor by IM MS. At moderate collision voltages, the IM plots show signals at similar CCS about 7630 Å^2^ for the in vitro and 7595 Å^2^ for the *in cellula*-formed complex. Noticeable is the comparably broad signal distribution for the in vitro complex as opposed to the *in cellula* dimers. Comparison of both IM plots is most intuitive in the difference spectrum of both IM plots (Fig. 10c, f). This implies that the *in cellula* assembled αβ heterodimer is present with a more homogenous structure, while the in vitro formed complexes seem less defined. The comparison of both complexes during the CIU experiment demonstrates that in vitro assembled αβ unfolds at a lower voltage than the *in cellula* formed heterodimer, validating that the *in cellula* formed complex is intrinsically more stable than the in vitro construct. These are interesting observations in the light of the subunit β’s ability to undergo four different conformations with different nucleotide-binding affinities, tight (T), loose (L), closed (C), and open (O) resulting in ATP hydrolysis/synthesis^46^. The stable and homogeneous conformation found for the *in cellula* complex might suggest a potentially chaperone-induced bias towards a single conformation, which supports assembly. In our, in vitro assembly assays no such bias towards a specific conformation is induced, explaining the differences in stability and hydrolysis activity. This structural difference propagates to the whole in vitro F_1_ which can be seen in the dependence on ATP/Mg^2+^ of the in vitro F_1_ complexes to prevent dissociation (Supplementary Fig. 2e and f), which is less of an issue for their *in cellula* counterparts (Supplementary Fig. 7). This will as well be the reason behind the much higher ATPase activity of the *in cellula* complexes. A precise investigation of the structural conformation of the subunits/ subcomplexes compared to *in cellula* complexes will be the focus of future studies.

What is known about the role of the ε subunit so far, is its relevance in the coupling of the soluble part F_1_ with the membrane-embedded F_O_ and its regulatory role in the inhibition of ATP hydrolysis activity^21,24,25,47^. Interestingly, our ATP activity assays revealed that isolated purified ε itself shows an ATP hydrolysis activity, which is surprisingly even higher than for subunit β. The ε-subunit in bacterial F_1_F_O_ is known to perform its regulatory function, by taking a hairpin or extended conformation^7,48^, and isolated subunit ε of *Bacillus subtilis* was found to be able to bind ATP^43^. Our results suggest that this extensive conformational change is driven by ATP hydrolysis.

All in all, we could show that the soluble F_1_ part can be reconstituted in vitro from the purified F_1_-subunits in presence of nucleotide and Mg^2+^. Our studies have provided an exciting overview of the step-by-step assembly via well-defined subcomplexes towards the full complex, with a preformed αβ heterodimer as an essential building block. Our results show the special roles, especially of subunits β and ε for the functional complex. Differences in the ATPase activity of the generated F_1_ complex generated in vitro and *in cellula* seem to be based on structural differences suggesting that bacterial chaperones and/or other cellular effects (i.e., molecular crowding) may not be needed for correct stoichiometric assembly of the ATPase but assist to fine-tune the assembly into a fully functional F_1_.

## Methods

### Cloning expression vectors of the *A. woodii* F_1_ ATP synthase

Using pKB3-His (a pET21a[+] vector derivative) as a master plasmid, which was kindly provided by Karsten Brandt (Müller laboratory^27^ at Goethe University), we performed PCR for cloning of deletions or point mutations and ligation independent cloning with the In-Fusion HD Cloning Kit (Takara Bio). For cloning of single genes encoding for subunits and subcomplexes of F_1_ (*atpHAGDC* for subunit δ, α, γ, β and ε) with an N- or C-terminal tag (StrepI- or His_6_-tag) were cloned into a commercially available pET21a(+)-vector (Novagen). All generated plasmids are listed in Supplementary Table 1. A list of primers, used for cloning the respective plasmids is given in Supplementary Table 2. Stellar competent cells (Takara Bio) were used for plasmid amplification. All constructs including mutations were verified by sequencing (Microsynth Seqlab).

### Purification of heterologous F_1_ ATP synthase subunits and subcomplexes of *A. woodii* containing a His_6_-tag or a Strep-tag

All constructs were transformed in *E. coli* BL21gold(DE3) competent cells (AgilentTechnologies) following the provided manual. Lysogeny Broth (LB) Agar (Lennox) transformation plates supplemented with 100 µg/mL ampicillin were used for the transformation of all constructs. Cell colonies were grown at 37 °C overnight and stored at 4 °C for up to 2 weeks. A single clone was picked to inoculate a pre-culture of LB containing the respective antibiotic and was grown at 37 °C and 150 rpm overnight. Pre-culture was inoculated in the main culture of Terrific Broth medium supplemented with respective antibiotics. Cells were cultivated at 37 °C and 150 rpm until an OD_600nm_ of 0.5–0.7 was reached. After inducing protein overexpression in the main culture with 0.25 mM Isopropyl-β-d-thiogalactopyranoside (IPTG) the protein expression was performed at 20 °C and 150 rpm overnight. The cells were harvested by centrifugation at 4000 × *g* for 20 min at 4 °C. The isolated cell pellets were resuspended in 20 mL wash buffer I (50 mM KP_i_, 200 mM NaCl, 20 mM imidazole, 10% (v/v) glycerol, pH 7.4) supplemented with 1 mM EDTA and DNAse I. Using a French Pressure Cell Press cells were mechanically disrupted at a pressure of 700 bar and cell debris were removed by centrifugation (50,000 × *g* for 60 min at 4 °C). After the addition of 2 mM MgCl_2_ cell lysate was incubated with 5 mL Ni-NTA Agarose (binding capacity 50 mg/mL) (MACHEREY-NAGEL) for 30 min. The column was washed with five column volumes wash buffer I to remove unbound proteins and bound protein was eluted with 2.5 column volumes wash buffer I containing 300 mM imidazole. The elution fractions were analyzed with (sodium dodecyl sulfate–polyacrylamide gel electrophoresis, concentrated with an Amicon Ultra centrifugal filter units, and purified by SEC with a Superdex 75 10/300 (GE-Healthcare). Protein samples were shock frozen in liquid nitrogen and stored at −80 °C. The same workflow procedure was performed for all protein constructs which contained a StrepI-tag with the exception of using a 5 mL Strep-Tactin-column (binding capacity 15 mg/mL resin) (Iba) with the respective wash buffer II (50 mM KP_i_, 200 mM NaCl, 10% (v/v) glycerol, pH 7.4) and elution with the wash buffer II containing 2.5 mM d-Desthiobiotin.

### In vitro assembly and mass spectrometric analysis

For the in vitro studies, all in *E. coli* heterologously purified subunits α, β, δ, and γε were incubated in a ratio of 3:3:1:1 for assembly into the full F_1_-complex, or subsets for subcomplexes in 50 mM KP_i_, 200 mM NaCl, 2 mM ATP, 2 mM MgCl_2_, 10% (v/v) glycerol, pH 7.4 at 4 °C for 1 h. For LILBID-MS measurements the buffer was exchanged to 100 mM ammonium acetate, 2 mM ATP, 2 mM MgCl_2_, pH 7.4 at 4 °C before measurements using desalting columns with a cutoff of 7 kDa (Zeba Micro Spin Desalting Columns, Thermo Scientific). Approximately 5 µL samples were used per measurement. LILBID-MS droplets were generated by a piezo-driven droplet generator (MD-K-130, Microdrop Technologies GmbH, Germany). This generator produces droplets of 50 µm diameter with a frequency of 10 Hz at 100 mbar. The droplets were transferred to a vacuum and irradiated by an IR laser at 2.94 µm, a vibrational absorption wavelength of water, which leads to the explosive expansion of the sample droplet. The energy output has a maximum energy of 23 mJ per pulse and a pulse length of 6 ns. The overall gradient between the first lens (repeller) and the third lens (grounded lens) is always 6.6 kV. The applied voltage on the second lense (extractor) is set between 4.0 kV and 6.0 kV. Either the first two lenses were pulsed from 0 kV to their final voltages, or both lenses were set at the same voltage of 4.0 kV and the repeller was pulsed up to 6.6 kV in total. The released ions then are analyzed using a home-built time-of-flight spectrometer^49^. These settings correspond to the standard settings at the LILBID ion source. The presented spectra show the averaged signals of several hundred up to thousand droplets. Data processing was done using the software Massign^50^.

Electrospray ionization mass spectrometry (ESI-MS) was performed on a Synapt G2S (Waters Corpn., Wilmslow, Manchester, UK) equipped with a high mass quadrupole upgrade. Pd/Pt sputtered nESI tips were pulled in-house from borosilicate glass capillaries on a Flaming/Brown Micropipette Puller (P-1000; Sutter Instrument Co.). For the analysis of purified F_1_-subunits, capillary and cone voltages were set to 1.85 kV and 150 V, respectively. The source block temperature was set to 30 °C. Neither trap nor transfer collision cell was used. Directly prior to MS analysis, 10 μL of the protein solution (30 µM) was buffer exchanged into 100 mM ammonium acetate, 100 µM ATP, 100 µM MgCl_2_, and pH 7.4 at 4 °C using Zeba Micro Spin Desalting Columns (Thermo Scientific).

For the IM MS experiments, αβ heterodimers were analyzed in positive ion mode using a capillary voltage of 2.1 kV. The rest of the settings for MS analysis were adjusted as follows: cone voltage 100 V at an offset of 150 V, 20 °C source temperature. The instrument was calibrated by a conventional CsI solution. IM experiments were done using a traveling wave setup operating at a wave height of 40 V, a traveling wave velocity of 700 m/s, a nitrogen gas flow of 90 mL/min, and a drift cell pressure of 3.5 mbar. MS-MS was performed to isolate the αβ heterodimers, which were selected at m/z = 4950 using an LM resolution of 12 and an HM resolution of 15. CIU experiments were performed by increasing the trap collision energy (CE) in steps of 5 V from 85 to 200 V. Data analysis of ESI-MS and IMS experiments was done using the software MassLynx V4.1, TWIMExtract^51^, CIUSuite^52^, and UniDec^53^. All mass spectra were normalized to a maximal intensity which is displayed in arbitrary units (a.u.).

### ATP hydrolysis assay

The enzyme-coupled activity assay performed in this work was adapted from references^41,42^. Generally, assays were run in 96-well f-bottom microtiter plates (Greiner Bio-One). The ATP hydrolysis assay was performed to analyze the ATP hydrolysis activity of the single F_1_-subunits, the in vitro assembled complexes of F_1_, and *in cellula* purified F_1_-subcomplexes. During this assay, ATP was constantly regenerated by an enzyme-coupled reaction, while the oxidation of NADH was spectroscopically followed via the decrease of absorbance at 340 nm. Measurements were performed in for subunits and in vitro assembled subcomplexes, for which the following amounts of the respective proteins were used: 5.5 µM in case of α and β, 1.8 µM γε, 5 µM ε. In the enzymatic assays of in vitro complexes 5.5 µM protein was used for α as well as β and 1.8 µM γε or 5 µM of the ε-subunit, respectively, to allow for subunit stoichiometries of 1:1 for the αβ heterodimer, and 3:3:1 for α_3_β_3_γε. *In cellula* produced αβ and α_3_β_3_γε* were employed with 0.5 µM and 0.25 µM, respectively.

Protein solutions were diluted to 100 µL total volume (100 mM TRIS, 100 mM maleic acid, pH 7.5, 5 mM MgCl_2_, 3 mM phosphoenolpyruvate, 4 mM ATP, 0.5 mM NADH, 10 units l-lactate dehydrogenase, 10 units pyruvate kinase), filtered and degassed for 700 s in 3 s intervals at 25 °C. The decrease in absorbance at 340 nm per second was determined by fitting a linear fit of the slope. All measurements were performed in biological triplicates except α_3_β_3_γε* with two technical duplicates. Kinetic data were analyzed with Origin 2018b and Excel 2016.

### HPLC analysis

For the assembly studies and chromatographic separations 60 µg protein was applied on an SEC column (bioZen 1.8 µm SEC-3) coupled with UV detection in the elution buffer A (50 mM KP_i_, 200 mM NaCl, pH 7.4) or elution buffer B (50 mM KP_i_, 200 mM NaCl, 2 mM ATP, 2 mM MgCl_2_, pH 7.4) at 4 °C, respectively.

### Reporting summary

Further information on research design is available in the Nature Research Reporting Summary linked to this article.

## Supplementary information

Supplementary Information

Reporting Summary

## Data Availability

The data generated in this study have been deposited in the Zenodo database and are available at 10.5281/zenodo.5865003. Source data are provided with this paper.

## Source data

Source data
