# Supplementary material for: Bacterial F-type ATP synthases follow a well-choreographed assembly pathway
Source: Nat Commun. 2022 Mar 8;13:1218. doi: 10.1038/s41467-022-28828-1 (PMC8904574; doi:10.1038/s41467-022-28828-1)
Supplement: Supplementary file 1 — Supplementary Information [file 41467_2022_28828_MOESM1_ESM.pdf]

Supplementary Information

Bacterial F-type ATP synthases follow a well-choreographed assembly pathway

Khanh Vu Huu[1], Rene Zangl[1], Jan Hoffmann[1], Alicia Just[1] & Nina Morgner[1]

Author affilitions

[1]Institute of Physical and Theoretical Chemistry, Goethe University Frankfurt/Main, Max-von-Laue-Str. 9, 60438 Frankfurt/Main, Germany

Table of contents:

Supplementary Figures:

Supplementary Figure 1: Effects of ATP/Mg^{2+} concentrations on MS-resolution and protein stability

Supplementary Figure 2: LILBID spectrum of *in cellula* produced αβ heterodimers in dependence of ATP/Mg^{2}

Supplementary Figure 3: Role of α mutants on the assembly process

Supplementary Figure 4. Deconvoluted nESI-MS-spectra of ATP-binding of different □□μυταντσ

Supplementary Figure 5: Mass spectrometric analysis with LILBID of *in vitro* assembly

Supplementary Figure 6: Dependence of δ-subunit assembly to the $α_3β_3γε$ pre-complex regarding to steric hindrance of affinity-tags and without nucleotide/Mg^{2+} environment

Supplementary Figure 7: Purification and quality control of F_1 and F_1-subcomplexes

Supplementary Figure 8: Expression of the subunits γ, ε and γε-complex in *E. coli* BL21gold(DE3) cells

Supplementary Figure 9: Mass spectrometric analysis of next neighbor interactions by LILBID-MS in a nucleotide/Mg^{2+} environment

Supplementary Figure 10. Ion mobility and mass spectrum of □□ ηετεροδιμερ

Supplementary Tables:

Supplementary Table 1: List of plasmids

Supplementary Table 2: List of primers

Supplementary Table 3: Calculated and experimental masses of F_1-subunits, F_1 and F_1-subcomplexes of *A. woodii*

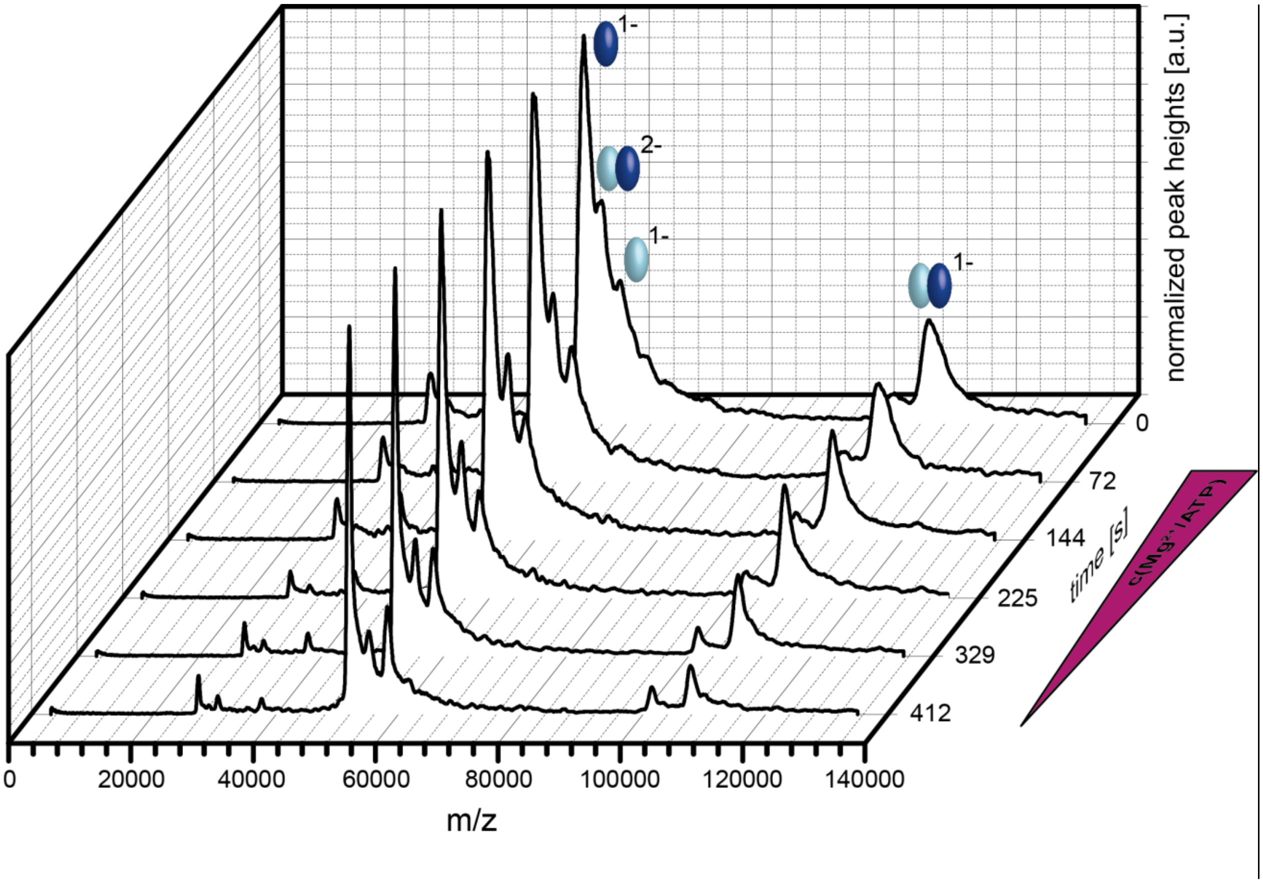

Supplementary Figure 1. Effects of ATP/Mg^{2+} concentrations on MS-resolution and protein complex stability. LILBID-spectra of the *in vitro* assembled α- (light blue) and β-subunit (dark blue) in 100 mM NH_4OAc, 2 mM ATP, 2 mM $MgCl_2$, pH 7.4. The LILBID droplet generator is filled with water before loading the sample of interest in front of the water. Over time the smaller molecules of the additives diffuse faster into the water than the proteins, leading to a dilution of the additives. Mass spectrometric measurements show the effects of this dilution, demonstrating that high ATP/Mg^{2+} concentrations are stabilizing the αβ heterodimer complex but as well decrease the mass resolution. Source data are provided as a Source Data file.

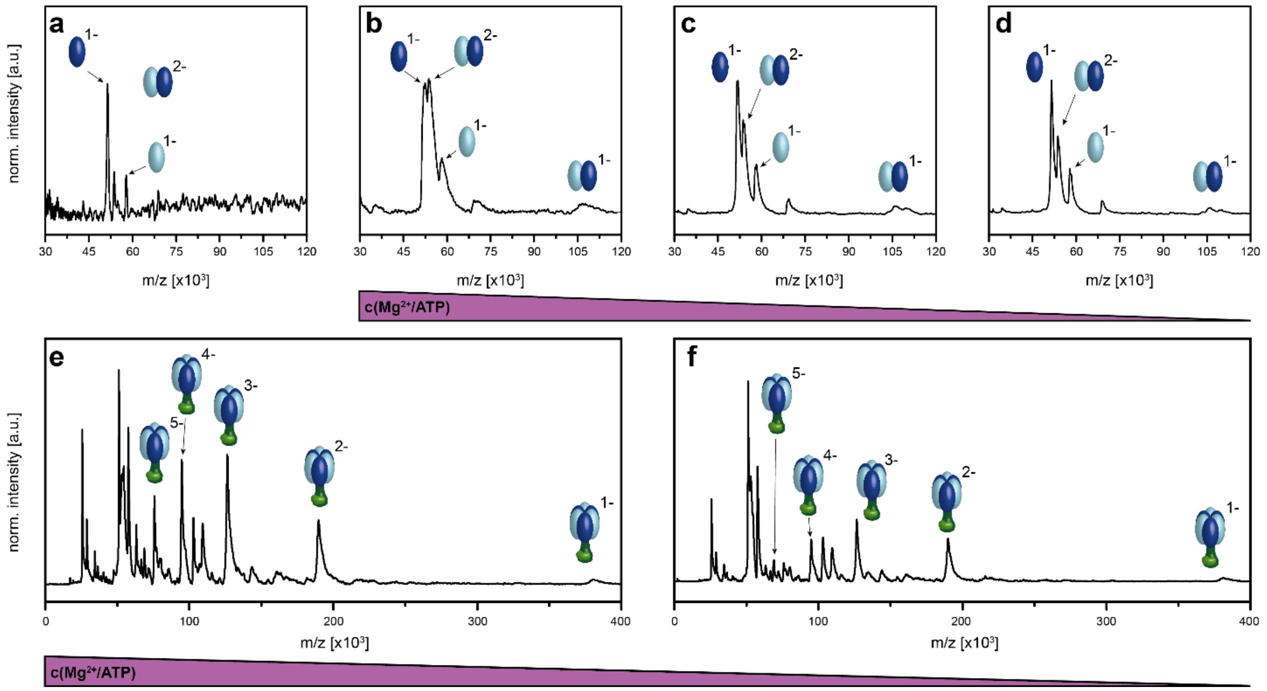

Supplementary Figure 2. LILBID spectra of *in cellula* produced αβ heterodimers in dependence of ATP/Mg^{2+}: a *In cellula* purified αβ heterodimer (light and dark blue). Mass spectrometric analysis shows that without ATP/Mg^{2+} the *in cellula* purified αβ heterodimer dissociates mostly into the subunits showing little stable complex. **b** *In cellula* purified αβ heterodimer (light and dark blue) in 2 mM ATP and 2 mM $MgCl_2$ shows a stabilization of the heterodimer accompanied by a decrease in signal resolution. **c** Buffer dilution leads to improved signal resolution. Comparison after 7 min (**d**) shows less dissociation of the dimer than the corresponding *in vitro* dimer (Supplementary Fig. 1) **e**-**f** *In vitro* assembled $\alpha_3\beta_3\gamma\varepsilon$ complexes dissociate due to the dilution of the additives ATP/Mg^{2+}. Source data are provided as a Source Data file.

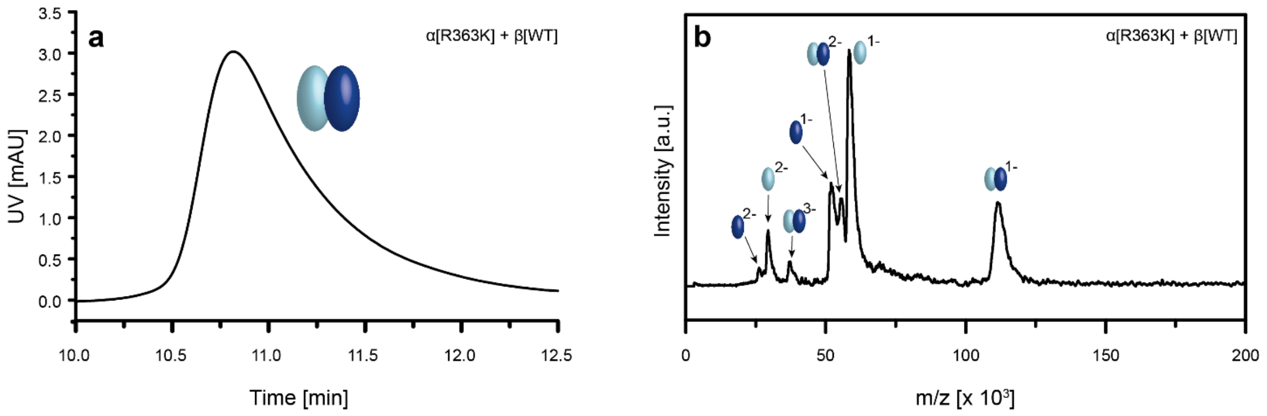

Supplementary Figure 3. *In vitro* assembled α[R363K] and β[WT]. a HPLC shows αβ heterodimer assembly at an elution peak of 10.86 min. Running buffer: 50 mM KP_i, 200 mM NaCl, 2 mM ATP, 2 mM $MgCl_2$, pH 7.4. **b** LILBID: The α- (light blue), β-subunit (dark blue) were incubated at 4 °C for 1 hour. Mass spectrometric analysis revealed αβ heterodimer assembly with a charge state distribution (-1 to -3). Exchange of the positively charged arginine to an as well positive lysine does not hamper formation of the heterodimer. Source data are provided as a Source Data file.

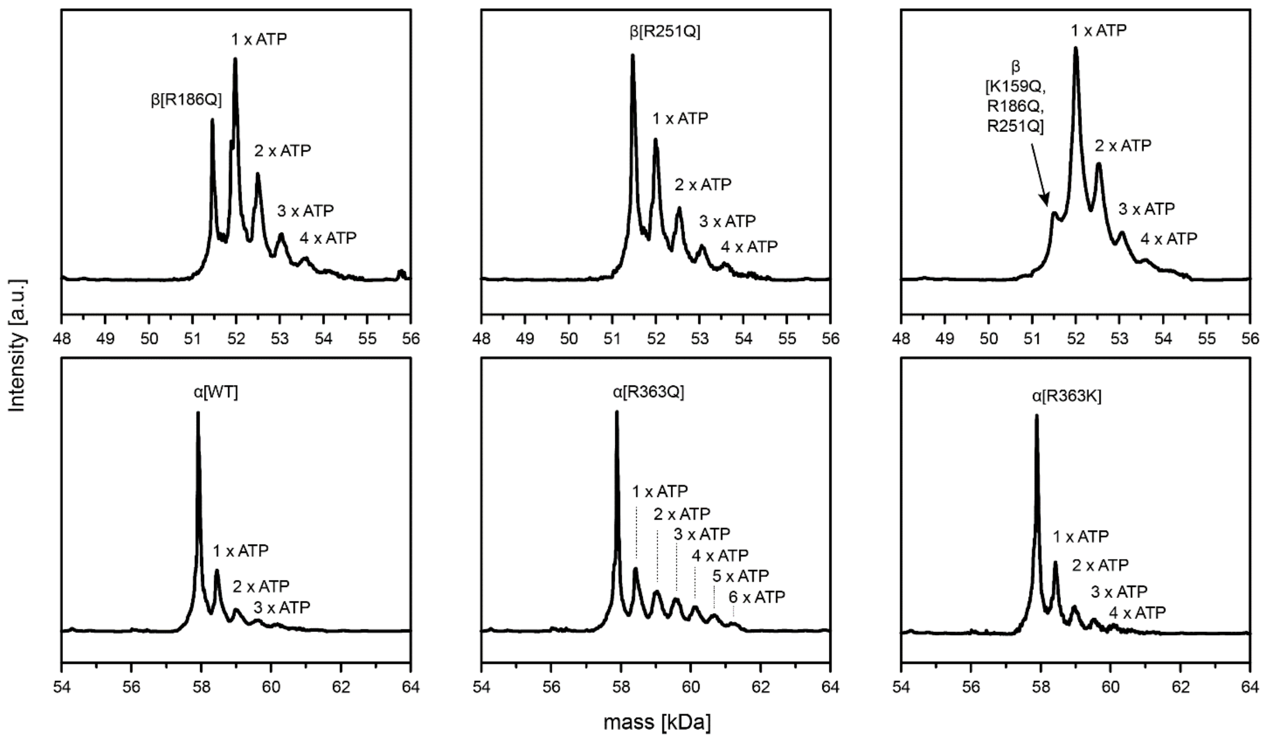

Supplementary Figure 4. Deconvoluted nESI-MS-spectra of ATP-binding of different β mutants. Source data are provided as a Source Data file.

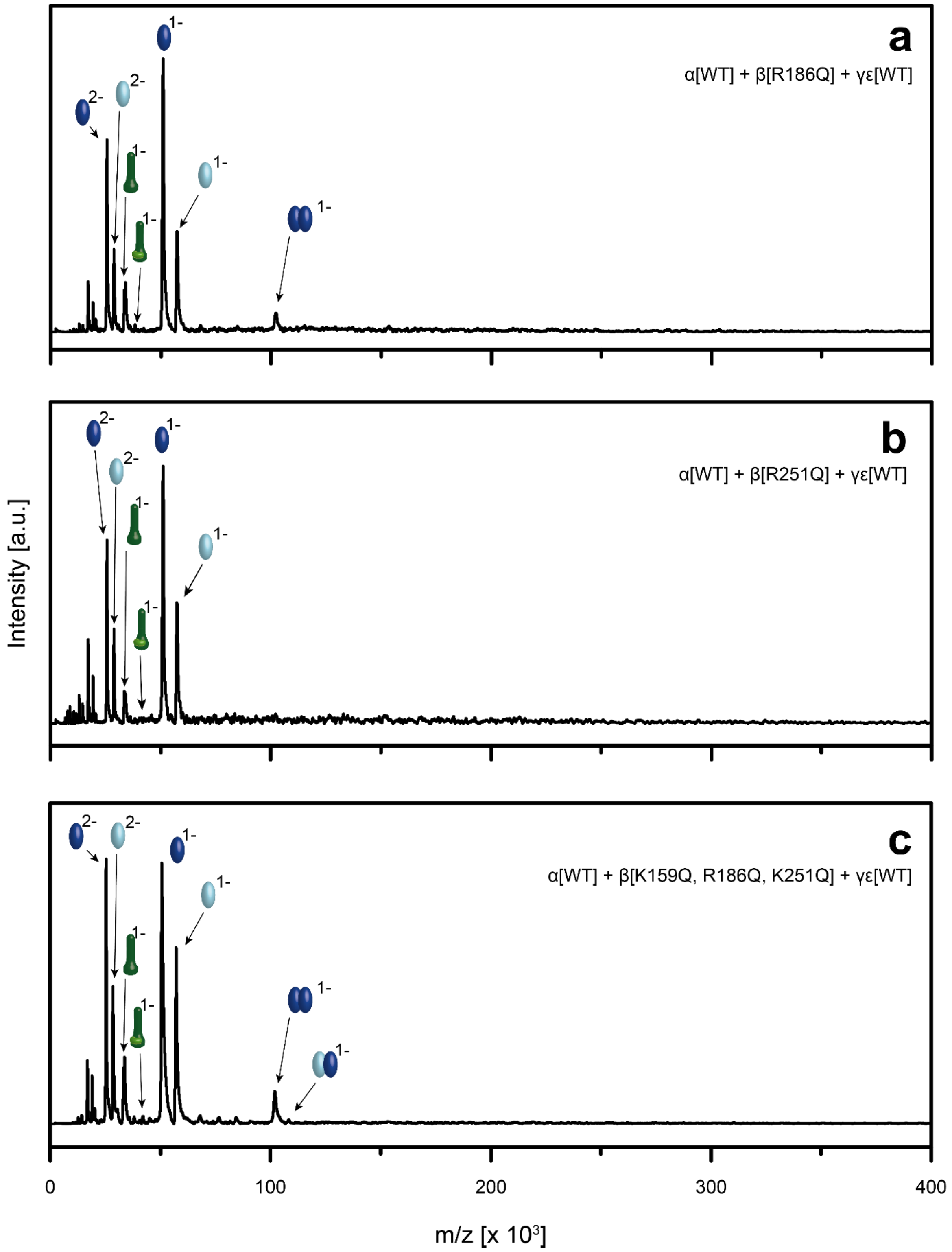

Supplementary Figure 5. Mass spectrometric analysis with LILBID of *in vitro* assembly of α[WT] and the central stalk γε with β mutants. *In vitro* assembly of α[WT] (light blue) and γε (dark and light green) with β[R186Q] (**a**) (dark blue), β[R251Q] (**b**) (dark blue) and β[K159Q, R186Q, R251Q] (**c**) (dark blue). Incubation in 50 mM KP_i, 200 NaCl, 20 mM imidazole, 10% (v/v) glycerol, pH 7.4 at 4 °C for 1 hour and finally buffer exchanged in 100 mM NH_4OAc, 2 mM ATP, 2 mM $MgCl_2$, pH 7.4. Source data are provided as a Source Data file.

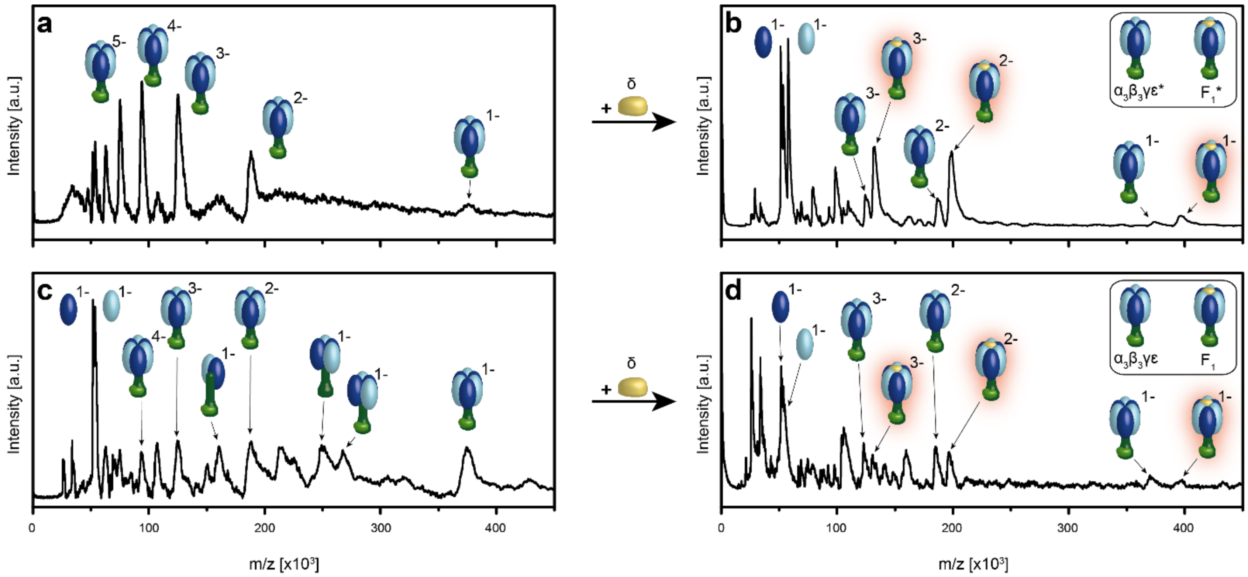

Supplementary Figure 6. δ-subunit binding to the *in cellula* purified $\alpha_3\beta_3\gamma\varepsilon$ pre-complexes as control for potential steric hindrance due to affinity-tags. All measurements were performed without nucleotide/Mg^{2+} environment. **a**-**b** LILBID-MS: *In vitro* assembly of the δ-subunit (yellow) with the $\alpha_3\beta_3\gamma\varepsilon^*$-complex. The in *E. coli* heterologously purified $\alpha_3\beta_3\gamma\varepsilon^*$ corresponds to the subcomplex which can be *in vitro* assembled by single subunits α (light blue), β (dark blue) and γε (dark and light green). The α- and β- subunits are N-terminally StrepI- and His_6-tagged, respectively. The ε-subunit is C-terminally His_6-tagged. **c**-**d** *In vitro* assembly of the δ-subunit (yellow) with the $\alpha_3\beta_3\gamma\varepsilon$ complex. This complex was only N-terminal His_6-tagged in the β- subunit. Our data show that the complex $\alpha_3\beta_3\gamma\varepsilon^*$ can be produced *in cellula* and the N-terminal affinity-tags in all α and β subunits in the $\alpha_3\beta_3\gamma\varepsilon^*$ complex have no steric effect that would hinder the assembly of the complex itself or the δ-subunit to the hexameric head. The full reconstitution into F_1 occurs here independently of nucleotides and Mg^{2+}. Interestingly, the subcomplexes $\alpha_3\beta_3\gamma\varepsilon^*$and $\alpha_3\beta_3\gamma\varepsilon$ can be isolated and purified *in cellula* from *E. coli* and form stable complexes without the presence of ATP/Mg^{2+} as opposed to our *in vitro* complexes. Source data are provided as a Source Data file.

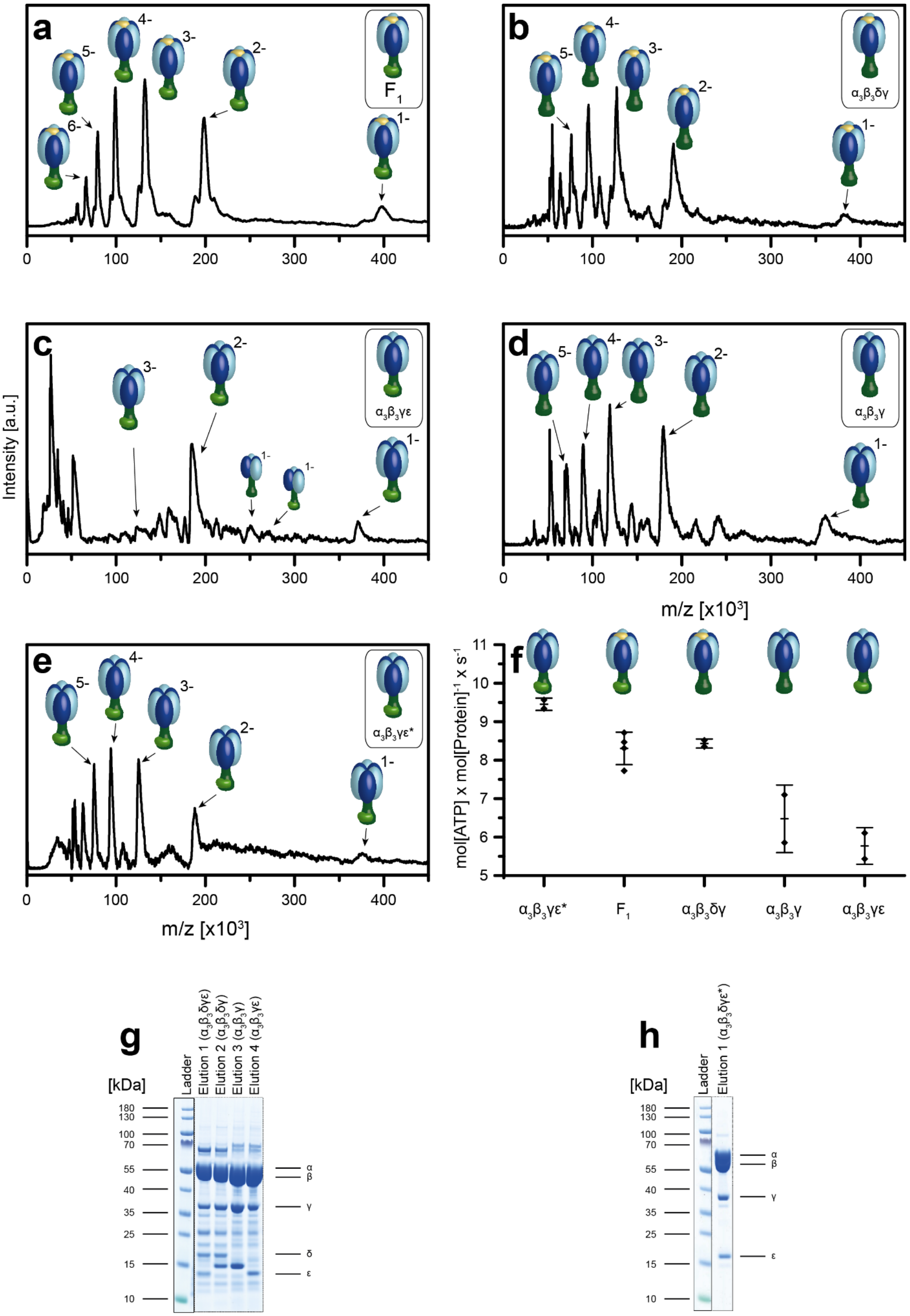

a
F1
6-
5-
4-
3-
2-
1-
b
α3β3δγ
c
α3β3γε
d
α3β3γ
e
α3β3γε*
Intensity [a.u.]
m/z [x10³]
0
100
200
300
400
f
mol[ATP] x mol[Protein]⁻¹ x s⁻¹
5
6
7
8
9
10
11
α3β3γε*
F1
α3β3δγ
α3β3γ
α3β3γε
g
[kDa]
Ladder
Elution 1 (α3β3δγε)
Elution 2 (α3β3δγ)
Elution 3 (α3β3γ)
Elution 4 (α3β3γε)
180
130
100
70
55
40
35
25
15
10
α
β
γ
δ
ε
h
[kDa]
Ladder
Elution 1 (α3β3γε*)
180
130
100
70
55
40
35
25
15
10
α
β
γ
ε

Supplementary Figure 7. Purification and quality control of *in cellula* obtained F_1 and F_1-subcomplexes. LILBID-MS spectrum of F_1 (**a**), $\alpha_3\beta_3\delta\gamma$ (**b**) $\alpha_3\beta_3\gamma\varepsilon$ (**c**) $\alpha_3\beta_3\gamma$ (**d**) and $\alpha_3\beta_3\gamma\varepsilon^*$ (**e**). **f** ATP hydrolysis activity of each complex. Data are presented as mean values with error bars show the SD, with individual data superimposed. Data were collected in biological duplicates (n = 2) except F_1 with four biological replicates (n = 4). **g** SDS-PAGE (NuPAGE 4-12% Bis-Tris) of Ni-chelating affinity chromatograpghy of purified F_1 and F_1-subcomplexes. All constructs were N-terminal His_6-tagged in the β-subunit. Isolation of purified F_1 ($\alpha_3\beta_3\delta\gamma\varepsilon$) complex (Elution 1), $\alpha_3\beta_3\delta\gamma$ (Elution 2), $\alpha_3\beta_3\gamma$ (Elution 3) and $\alpha_3\beta_3\gamma\varepsilon$ (Elution 4). N = 3 independent experiments. **h** SDS-PAGE (NuPAGE 4-12% Bis-Tris) of tandem affinity chromatography of purified F_1-subcomplex ($\alpha_3\beta_3\gamma\varepsilon^*$), using Ni-chelating followed by Strep-Tactin chromatography. This in *E. coli* heterologous purified $\alpha_3\beta_3\gamma\varepsilon^*$ complex is N-terminal StrepI- and His_6-tagged in α and β, respectively, where the ε-subunit is C-terminal His_6-tagged. N = 3 independent experiments. Source data are provided as a Source Data file.

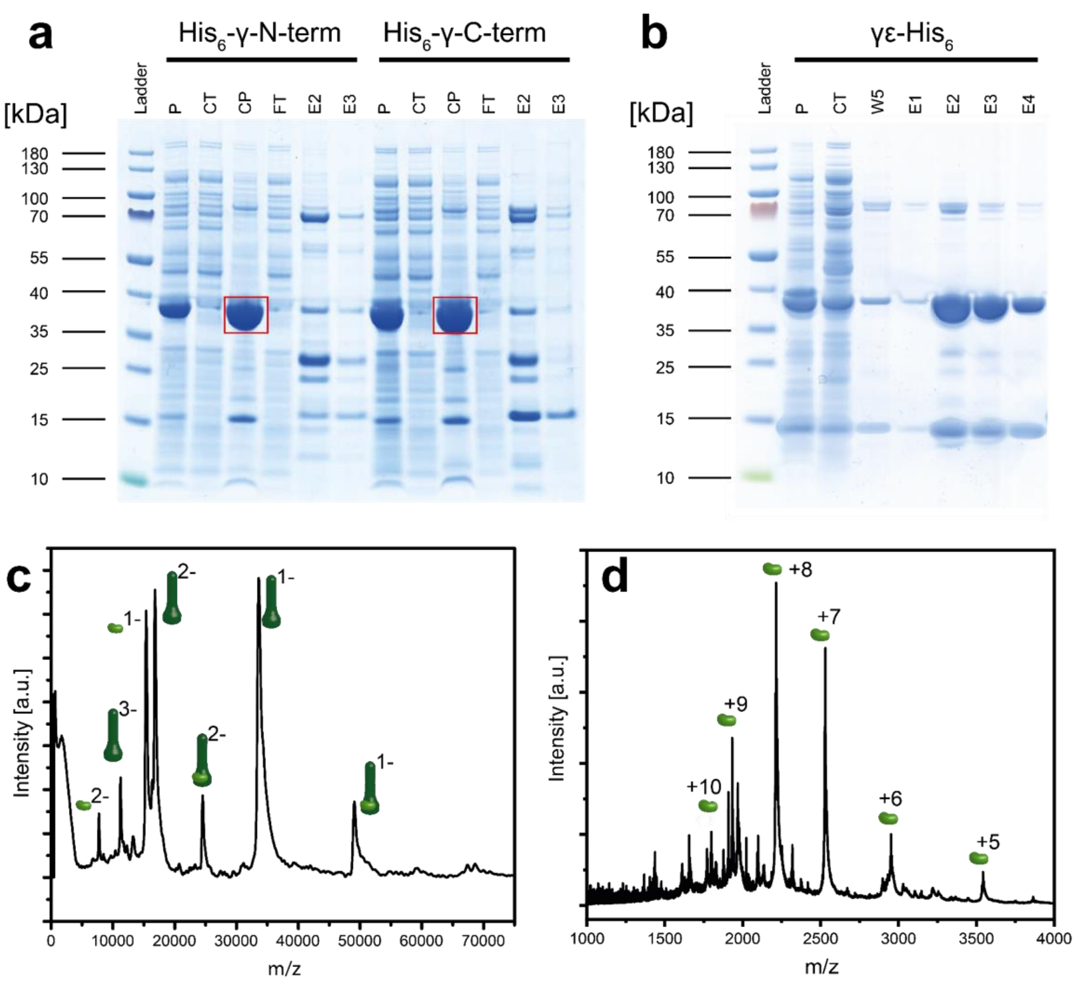

Supplementary Figure 8. Expression of the subunits γ, ε and γε-complex in *E. coli* BL21gold(DE3) cells. a Dependence of the affinity tag (His_6-tag) at the N- or C-terminus. In both cases the γ-subunit forms inclusion bodies in the cell pellet (CP) (indicated in red boxes). Abbreviations: pellet (P), cytoplasm (CT), flow through (FT), elution fraction 2 (E2) and elution faction 3 (E3). N = 3 independent experiments. **b** Coomassie-stained SDS-PAGE shows successful purification of the γε complex (harboring a C-terminal His_6- or StrepI-tag) indicated in elution fractions 1-4 (E1-4). **c** Quality control via LILBID-MS shows the γε complex with a charge state distribution (marked with -1 and -2). Due to

high laser energy the complex dissociated partially into γ (dark green) and ε (light green). **d** nESI spectrum of the ε-subunit (containing a C-terminal StrepI-tag) with a charge state distribution (+5 to +10) could verify successful purification. N = 3 independent experiments. Source data are provided as a Source Data file.

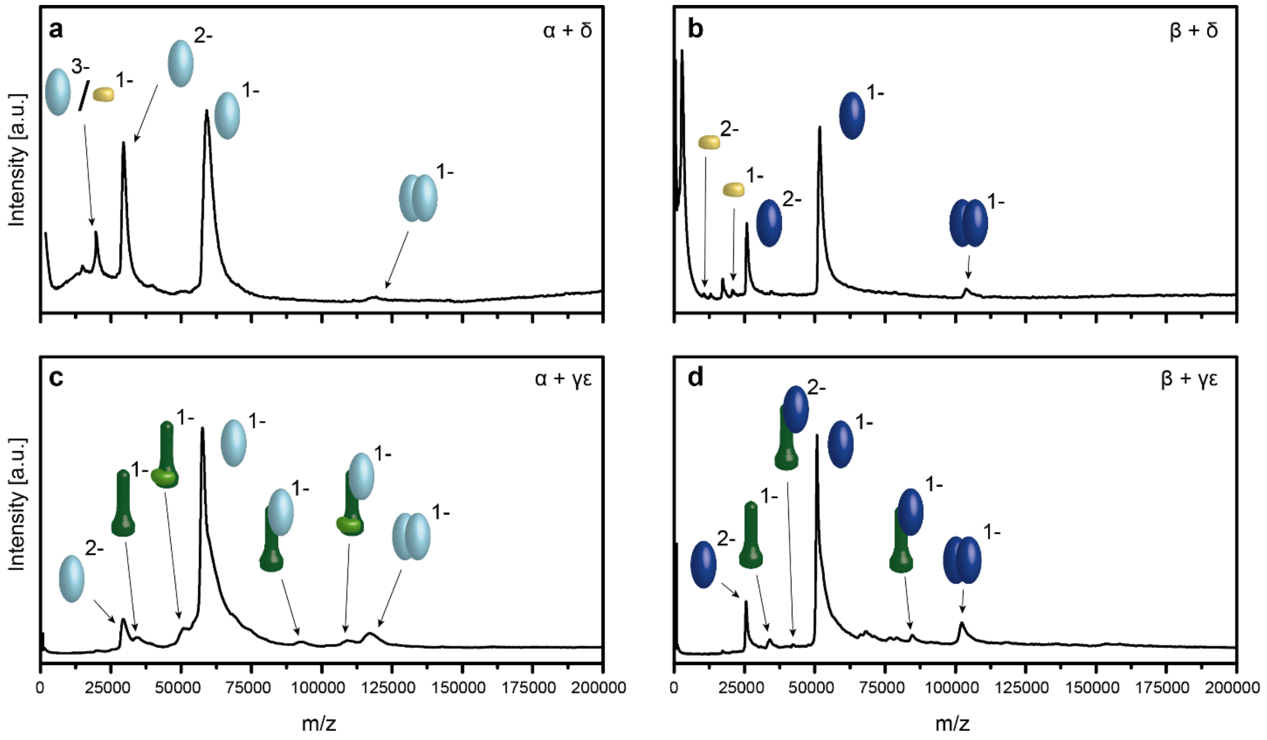

Supplementary Figure 9. Mass spectrometric analysis of next neighbor interactions by LILBID-MS in a nucleotide/Mg^{2+} environment. All incubation experiments were done at protein ratios as expected from the complexes (α:δ in a ratio 3:1) **a**-**b** Assembly studies of subunit α (light blue) or β (dark blue) with δ demonstrate no specific interactions into higher complexes. **c**-**d** Assembly studies of subunit α (light blue) or β (dark blue) with the central stalk γε show a binding ratio of 1:1. Due to high laser energy the complexes dissociated partially into αγ or βγ and dissociated subunit ε. Source data are provided as a Source Data file.

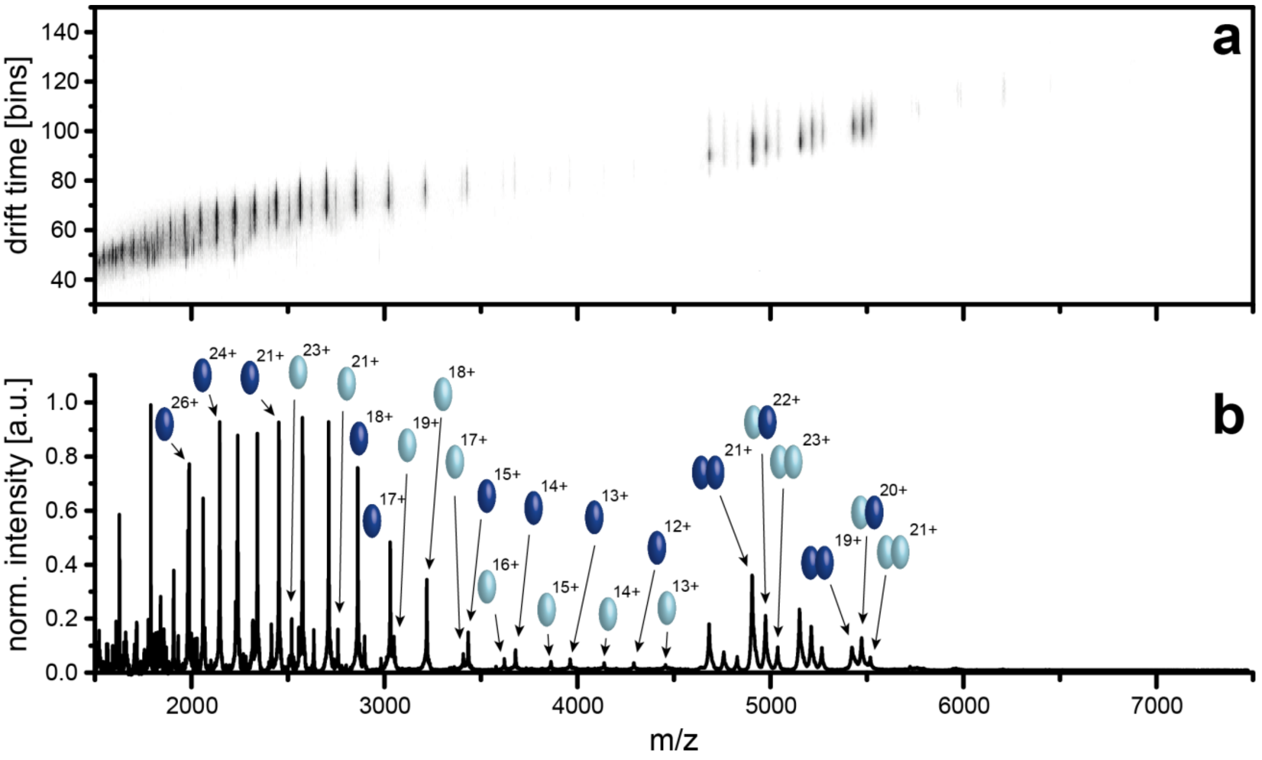

Supplementary Figure 10. Ion mobility and mass spectrum of αβ heterodimer. **a** Ion mobility. **b** mass spectrum. Source data underlying Supplementary Fig. 10b are provided as a Source Data file.

Supplementary Table 1. List of plasmids.

	Plasmid-Nr.	Construct	Name
**1.**	pKV006	β	pKV006_pET21a_His6-atpD_N-term
**2.**	pKV007	α	pKV007_pET21a_StrepI-atpA_N-term
**3.**	pKV025	ε	KV025_pET21a_atpC-StrepI-C-term
**4.**	pKV026	ε	KV026_pET21a_atpC-His6-C-term
**5.**	pKV027	γε	pKV027_pET21a_atpG-atpC-His6-C-term
**6.**	pKV030	$\alpha_3\beta_3\gamma\varepsilon^*$	pKV030_pET21a_tetracis(StrepI-atpA-N-term_His6-atpD-N-term_atpG_atpC-His6-C-term)
**7.**	pKV032	δ	pKV032_pET21a_atpH-His6-C-term
**8.**	pKV035	β[K159Q]	pKV035_pET21a_His6-atpD_[K159Q]
**9.**	pKV036	β[R186Q]	pKV036_pET21a_His6-atpD_[R186Q]
**10.**	pKV037	β[R251Q]	pKV037_pET21a_His6-atpD_[R251Q]
**11**	pKV038	β[K159Q_R186Q_R251Q]	pKV038_pET21a_His6-atpD_[K159Q_R186Q_R251Q]
**12.**	pKV043	αβ	KV043_pET21a_bicis(StrepI-atpA_His6-atpD-N-term)
**13.**	pKV044	$\alpha_3\beta_3\delta\gamma\varepsilon$	pKV044_pET21a-atpH-atpA-atpG_His6-N-term_atpD_atpC
**14.**	pKV045	$\alpha_3\beta_3\delta\gamma$	pKV045_pET21a_tetracis(atpH_atpA_His6-atpD-N-term_atpG)
**15.**	pKV046	$\alpha_3\beta_3\gamma$	pKV046_pET21a_tricis(atpA_His6-atpD-N-term_atpG)
**16.**	pKV047	$\alpha_3\beta_3\gamma\varepsilon$	pKV047_pET21a_tetracis(atpA_His6-atpD-N-term_atpG_atpC)
**17.**	pKV050	Δα[R363Q]	pKV050_pET21a_StrepI-atpA_[R363Q]
**18.**	pKV051	Δα[R363K]	pKV051_pET21a_StrepI-atpA_[R363K]

Supplementary Table 2. List of primer.

Number	Name	Length	Orientation	T_m [°C]	Sequence [5'3']	Construct Template
**KV001**	His6-atpD_N-term_for	38	forward	53.5	CACCATCACCATCACGCCCAAAATATAGGGAAGGTTGT	pKV006
**KV054**	His6-atpD-N-term_rev	44	reverse	53	GAATTCGGATCCCTAACCTTTGATTTTTTTTGCTTTTACTACAG	pKV006
**KV011**	StrepI-atpA-N-term	78	forward	54	GGTTCTGGCGGTGGATCGGGAGGTTCAGCGTGGAGCCACCCGCAGTTCGAAAAAAATCTCCGACCAGAGGAAATAAGT	pKV007
**KV052**	StrepI-atpA-N-term_rev	48	reverse	55.7	GAATTCGGATCCCTATACAGATTTACTGAAAACTTTTTTATAAGCCTC	pKV007
**KV058**	atpG-atpC_for	24	forward	54	GAAGGAGATATACATATGGCAGAG	pKV027
**KV047**	atpG-atpC-His6-C-term_rev	41	reverse	53.2	ATGGTGATGGTGATGTACATTTTCTTTTGAGTTGATCCGAG	pKV027
**KV011**	StrepI-atpA-N-term	78	forward	54	GGTTCTGGCGGTGGATCGGGAGGTTCAGCGTGGAGCCACCCGCAGTTCGAAAAAAATCTCCGACCAGAGGAAATAAGT	pKV030
**KV097**	atpG-atpC-His6_rev	39	reverse	55.7	GCAAGCTTGTCGACGCTAGTGATGGTGATGGTGATGTAC	pKV030
**KV075**	atpH-His6_for	42	forward	55.2	GAAGGAGATATACATATGAGTTTAGTTGCAAGTAAATACGCC	pKV032
**KV076**	atpH-His6_rev	46	reverse	53.8	ATGGTGATGGTGATGATGTAGTCTTAAATTGTTCACTTGTTTTTTC	pKV032
**KV079**	atpD_[K159Q]_for	37	forward	54.8	CAGACCGTATTGATTCAGGAATTAATTAATAATATTG	pKV035
**KV080**	atpD_[K159Q]_rev	25		53.5	AATCAATACGGTCTGACCAACTCCG	pKV035
**KV081**	atpD_[R186Q]_for	21	forward	55.9	CAGACCCGTGAAGGGAATGAC	pKV036
**KV082**	atpD_[K186Q]_rev	20	reverse	55.4	CCCTTCACGGGTCTGTTCTC	pKV036
**KV083**	atpD_[R251Q]_for	29	forward	54.8	CAGTTTACTCAAGCTGGTTCAGAAGTTTC	pKV037
**KV084**	atpD_[K251Q]_rev	31	reverse	54.4	AGCTTGAGTAAACTGGAAAATGTTATCAATG	pKV037
**KV083**	atpD_[R251Q]_for	29	forward	54.8	CAGTTTACTCAAGCTGGTTCAGAAGTTTC	pKV038
**KV084**	atpD_[K251Q]_rev	31	reverse	54.4	AGCTTGAGTAAACTGGAAAATGTTATCAATG	pKV038
**KV011**	StrepI-atpA-N-term	78	forward	54	GGTTCTGGCGGTGGATCGGGAGGTTCAGCGTGGAGCCACCCGCAGTTCGAAAAAAATCTCCGACCAGAGGAAATAAGT	pKV043
**KV090**	StrepI-atpA_His6-atpD_rev	47	reverse	55.4	GCAAGCTTGTCGACGCTAACCTTTGATTTTTTTTGCTTTTACTACAG	pKV043
**KV075**	atpH-His6_for	42	forward	55.2	GAAGGAGATATACATATGAGTTTAGTTGCAAGTAAATACGCC	pKV044
**KV099**	atpH_atpA_atpG_atpD_atpC_rev	26	reverse	53.2	TACATTTTCTTTTGAGTTGATCCGAG	pKV044
**KV075**	atpH-His6_for	42	forward	55.2	GAAGGAGATATACATATGAGTTTAGTTGCAAGTAAATACGCC	pKV045
**KV101**	atpH_atpA_atpG_His6-atpD_rev	45	reverse	53.4	TGCGGCCGCAAGCTTCTAACCTTTGATTTTTTTTGCTTTTACTAC	pKV045
**KV102**	atpA_atpG_His6-atpD_rev	41	forward	56	GAAGGAGATATACATATGAATCTCCGACCAGAGGAAATAAG	pKV046
**KV101**	atpH_atpA_atpG_His6-atpD_rev	45	reverse	53.4	TGCGGCCGCAAGCTTCTAACCTTTGATTTTTTTTGCTTTTACTAC	pKV046
**KV102**	atpA_atpG_His6-atpD_rev	41	forward	56	GAAGGAGATATACATATGAATCTCCGACCAGAGGAAATAAG	pKV047
**KV099**	atpH_atpA_atpG_atpD_atpC_rev	26	reverse	53.2	TACATTTTCTTTTGAGTTGATCCGAG	pKV047
**KV110**	atpA_[R363Q]_for	23	forward	53	GGGATTTCAGTATCTCAAGTTGG	pKV050
**KV111**	atpA_[R363Q]_rev	24	reverse	55.7	AGATACTGAAATCCCCGGGTTAAC	pKV050
**KV112**	atpA_[R363K]_for	25	forward	53	GGGATTTCAGTATCTAAGGTTGGTG	pKV051
**KV111**	atpA_[R363Q]_rev	24	reverse	55.7	AGATACTGAAATCCCCGGGTTAAC	pKV051

Supplementary Table 3. Calculated and experimental masses of F_1-subunits, F_1 and F_1-subcomplexes of *A. woodii*. The purified constructs bearing a N-terminal His_6- (0.978 kDa) or StrepI-tag (3.176 kDa) or a C-terminal His_6- or StrepI-tag. Errors shown are FWHM. (†) Affinity-tag is located at the C-terminus. (††) Affinity-tag is located at the N-terminus.

Construct	Molecular weight [kDa]			
	Calculated mass		Experimental mass	
	His_6-tag	StrepI-tag	His_6-tag	StrepI-tag
$\alpha^{\dagger\dagger}$	-	58.046	-	58 ± 2
$\beta^{\dagger\dagger}$	51.486	-	51.4 ± 1.7	-
$\varepsilon^{\dagger}$	15.660	17.864	15.4 ± 0.4	17.7 ± 0.2
$\delta^{\dagger}$	21.537	-	21.5 ± 0.5	-
$\gamma\varepsilon^{\dagger}$	49.676	-	49.1 ± 0.6	-
$\alpha_3\beta_3^{\dagger\dagger}\delta\gamma\varepsilon$ (F_1)	390.461	-	391 ± 15	-
$\alpha_3\beta_3^{\dagger\dagger}\delta\gamma$	375.624	-	375 ± 19	-
$\alpha_3\beta_3^{\dagger\dagger}\gamma\varepsilon$	369.801	-	370 ± 16	-
$\alpha_3\beta_3^{\dagger\dagger}\gamma$	358.910	-	359 ± 15	-
$\alpha_3^{\dagger\dagger}\beta_3^{\dagger\dagger}\gamma\varepsilon^{\dagger}$ ($\alpha_3\beta_3\gamma\varepsilon^*$)	378.272		378 ± 16	
